# Visualization of two architectures in class-II CAP-dependent transcription activation

**DOI:** 10.1371/journal.pbio.3000706

**Published:** 2020-04-20

**Authors:** Wei Shi, Yanan Jiang, Yibin Deng, Zigang Dong, Bin Liu

**Affiliations:** 1 Section of Transcription and Gene Regulation, The Hormel Institute, University of Minnesota, Austin, Minnesota, United States of America; 2 Section of Cellular and Molecular Biology, The Hormel Institute, University of Minnesota, Austin, Minnesota, United States of America; 3 Department of Pathophysiology, School of Basic Medical Sciences, Zhengzhou University, Zhengzhou, P.R. China; 4 Section of Cell Death and Cancer Genetics, The Hormel Institute, University of Minnesota, Austin, Minnesota, United States of America

## Abstract

Transcription activation by cyclic AMP (cAMP) receptor protein (CAP) is the classic paradigm of transcription regulation in bacteria. CAP was suggested to activate transcription on class-II promoters via a recruitment and isomerization mechanism. However, whether and how it modifies RNA polymerase (RNAP) to initiate transcription remains unclear. Here, we report cryo–electron microscopy (cryo-EM) structures of an intact *Escherichia coli* class-II CAP-dependent transcription activation complex (CAP-TAC) with and without de novo RNA transcript. The structures reveal two distinct architectures of TAC and raise the possibility that CAP binding may induce substantial conformational changes in all the subunits of RNAP and transiently widen the main cleft of RNAP to facilitate DNA promoter entering and formation of the initiation open complex. These structural changes vanish during further RNA transcript synthesis. The observations in this study may reveal a possible on-pathway intermediate and suggest a possibility that CAP activates transcription by inducing intermediate state, in addition to the previously proposed stabilization mechanism.

## Abstract 2

Cryo-EM structures of transcription activation complexes comprising cyclic AMP receptor protein (CAP) bound to a class-II promoter reveal two distinct architectures and suggest a possibility that CAP activates transcription by inducing an intermediate state, an important supplement to the previously proposed stabilization mechanism.

## Introduction

Transcription, the first step of gene expression, is regulated by various transcription factors. How these transcription factors modulate RNA polymerase (RNAP) is significant for understanding the mechanisms of transcription and gene regulation. In bacteria, cyclic AMP (cAMP) receptor protein (CAP) is a classic dimeric global transcription factor, which is activated by its allosteric effector cAMP [1–3]. CAP activates and initiates transcription on more than 100 promoters in *E*. *coli* [1], mainly via two different mechanisms: class-I and class-II, according to its interaction mode with RNAP holoenzyme [4–6], the main enzyme comprising a five-subunit core enzyme (α_2_ββ′ω) and a sigma factor, which is responsible for RNA synthesis in cells [7,8]. On class-I promoters, such as *lac* promoter, CAP binds at the −61.5 site of the promoter and interacts with RNAP holoenzyme by the carboxyl-terminal domain of the alpha subunit (αCTD) of RNAP and activates transcription via a recruitment mechanism [9,10]. On class-II promoters, as exemplified by *gal* promoter, CAP binds at the −41.5 site of the promoter and makes contact with multiple subunits of RNAP holoenzyme and initiates transcription via a recruitment and isomerization mechanism [4,11–15].

Previous electron microscopy structure of the class-I CAP-dependent transcription activation complex (CAP-TAC) [16] and our recent cryo–electron microscopy (cryo-EM) structure of the *E*. *coli* class-I CAP-TAC [17] have shown the overall architecture of the class-I complex and are consistent with the recruitment mechanism via simple stabilization of initiation complex on class-I promoters. Recent crystal structure of the *Thermus Thermophilus* class-II TAP (a CAP homolog in *T*. *thermophilus*)–dependent TAC (TAP-TAC) [18] has displayed an architecture in which there are no apparent CAP binding–induced conformational changes in RNAP, and consequently also suggested a simple stabilization mechanism for class-II activation. However, considering the extensive contact interface between CAP dimer and RNAP holoenzyme in the class-II TAC, it is possible that there are the CAP binding–induced conformational changes in RNAP, which are difficult to be captured by X-ray crystallography due to crystal packing but accessible in electron microscopy. Thus, the need for investigating the mechanism of class-II activation still remains.

In this study, we have determined three cryo-EM structures of the intact *E*. *coli* class-II CAP-TAC at around 4.3–4.5 Å resolutions, containing a CAP dimer, a σ^70^-RNAP holoenzyme, a complete class-II CAP-dependent promoter DNA, and with or without a de novo synthesized RNA oligonucleotide, and revealed two distinct architectures of the class-II TACs: state 1 and state 2. The state 1 architecture visualizes a complex that possibly could be an on-pathway intermediate in CAP-dependent transcription activation, raising the possibility that CAP binding might induce major conformational changes in RNAP and lead to a widening of the main cleft, which then might facilitate the entry of DNA promoter into the cleft and further isomerization into the RNAP-promoter open complex (RPo). On the basis of these structures, we suggest that, at class-II promoters, CAP may induce or stabilize an intermediate state with an open cleft in addition to stabilizing a final state.

## Results and discussion

### Overall structures of the *E*. *coli* class-II CAP-TACs without RNA transcript

**Fig 1:**
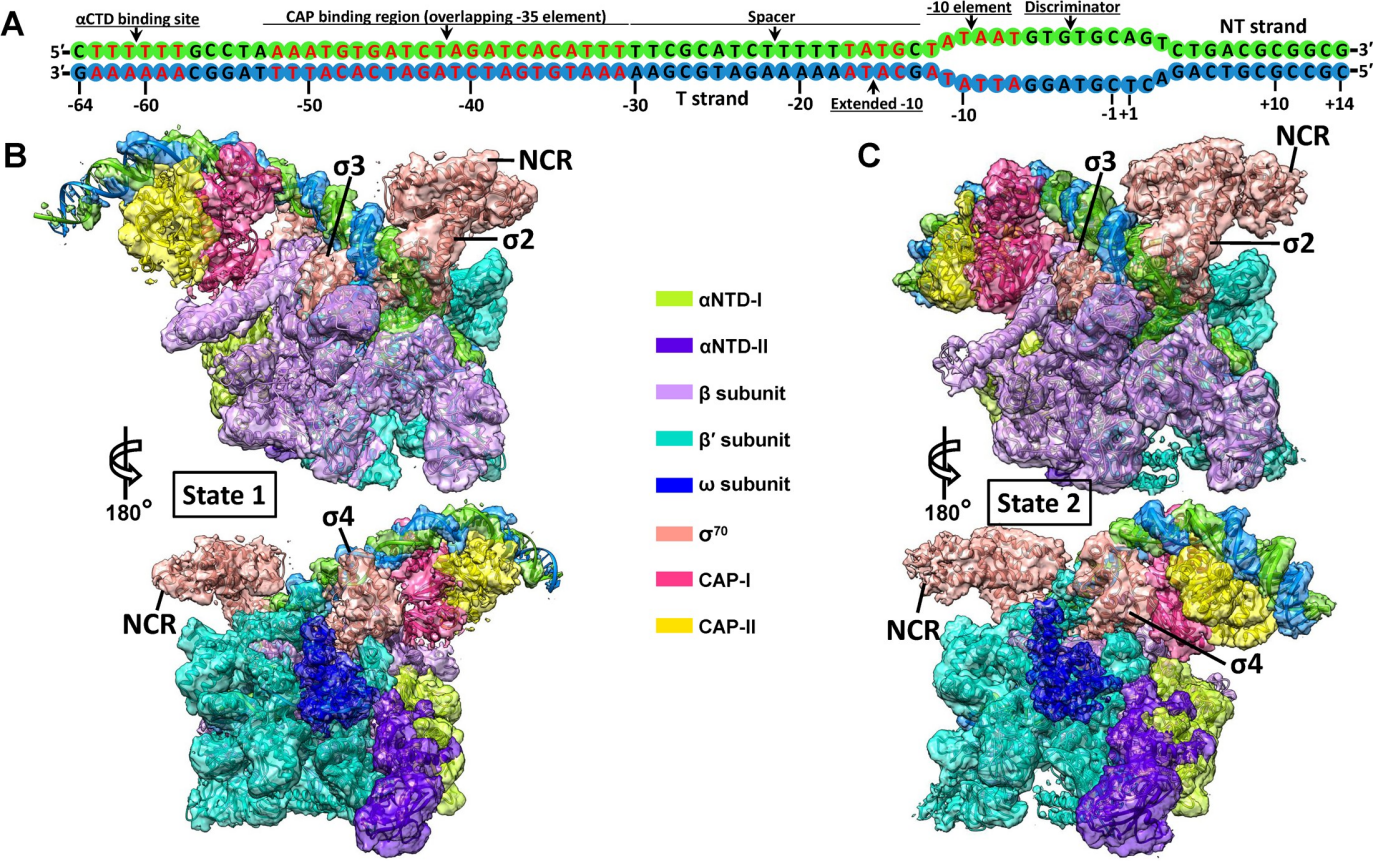
Cryo-EM reconstructions of the class-II CAP-TAC without RNA transcript. (A) Schematic representation of the synthetic promoter DNA scaffold (78 bp) in the class-II CAP-TAC. (B-C) Overviews of the cryo-EM reconstruction maps of the *E*. *coli* class-II CAP-TAC without RNA transcript at 4.5 Å (B, state 1) and 4.3 Å (C, state 2) resolutions, respectively. The individually colored density maps, created by color zone, split in Chimera, and shown in a contour of 8 root-mean-square (RMS), are displayed in transparent surface representation to allow visualization of all the components of the complex. CAP-TAC, CAP-dependent transcription activation complex; cryo-EM, cryo–electron microscopy; NCR, non-conserved region; NT, non-template; αCTD, carboxyl-terminal domain of the alpha subunit; αNTD, amino-terminal domain of the alpha subunit.

To investigate whether and how CAP binding induces conformational changes of RNAP to activate transcription on class-II promoters, a synthetic DNA scaffold representing the complete class-II CAP-dependent promoter (from −64 to +14, 78 bp in total) as observed in the *gal* promoter (Fig 1A) was designed to assemble the intact *E*. *coli* class-II CAP-dependent TAC in vitro (S1 Fig). The pre-open discriminator region (6 mismatched bases) on the DNA scaffold was designed to facilitate the further formation of a whole DNA bubble, which has been generally used in previous structural studies [17,19,20]. To test the effect of RNA transcript synthesis on the overall architectures, the complex was also assembled in the presence of the nucleotides (ATP and GTP) to synthesize RNA transcript.

The isolated complexes were subjected to cryo-EM analyses. The cryo-EM single-particle reconstructions of the intact class-II CAP-TAC without nucleoside triphosphate (NTP) incubation showed two different architectures of the structure: state 1 and state 2 at overall resolutions of 4.3 Å and 4.2 Å, respectively (S2 Fig and S1 Table). In the cryo-EM maps, the densities for RNAP holoenzyme allowed the unambiguous docking of its components, while the CAP-binding region is poorly defined due to high structural flexibility and low occupancy. The αCTD of RNAP is invisible in density maps, although a specific DNA sequence for its binding is included in the DNA scaffold. The non-conserved region (NCR) of σ^70^ and the ω subunit of the holoenzyme are visible at a low contour level, owing to their structural flexibilities and low occupancies. Further focused classifications and refinements with a sphere covering only the CAP-binding region improved the density for this portion and generated final overall 4.5 Å and 4.3 Å resolution maps for the state 1 and state 2 structures, respectively (Fig 1B and 1C). Three-dimensional (3D)–Fourier shell correlation (FSC) calculations showed a minor preferred orientation problem in both maps, but their half-map sphericity values (0.840 and 0.824, respectively) suggested no concerns on the directional resolution anisotropy issue (S3 and S4 Figs). In the state 1 structure, further local resolution map showed the almost invisible density on the DNA portion for αCTD binding, the presence of gaps in the DNA part that is next to the αCTD binding portion, and the relatively weak density in the parts of CAP dimer close to these gaps and the β flap tip (β-FT). All these suggested high flexibility of these regions in the CAP-binding region of the state 1 architecture, making it hard to specifically define the CAP-RNAP interface. However, the relatively stable major part of CAP dimer and its bound DNA and the clear density of RNAP apparently displayed the relative position between CAP dimer and RNAP, the upstream DNA path, and the distinct architecture of RNAP (S5A Fig). Further local resolution mapping of the state 2 structure showed the good density on both the CAP-binding region and RNAP and clearly displayed the CAP-RNAP interface (S5B Fig).

### Two distinct architectures reveal substantial conformational changes in RNAP

**Fig 2:**
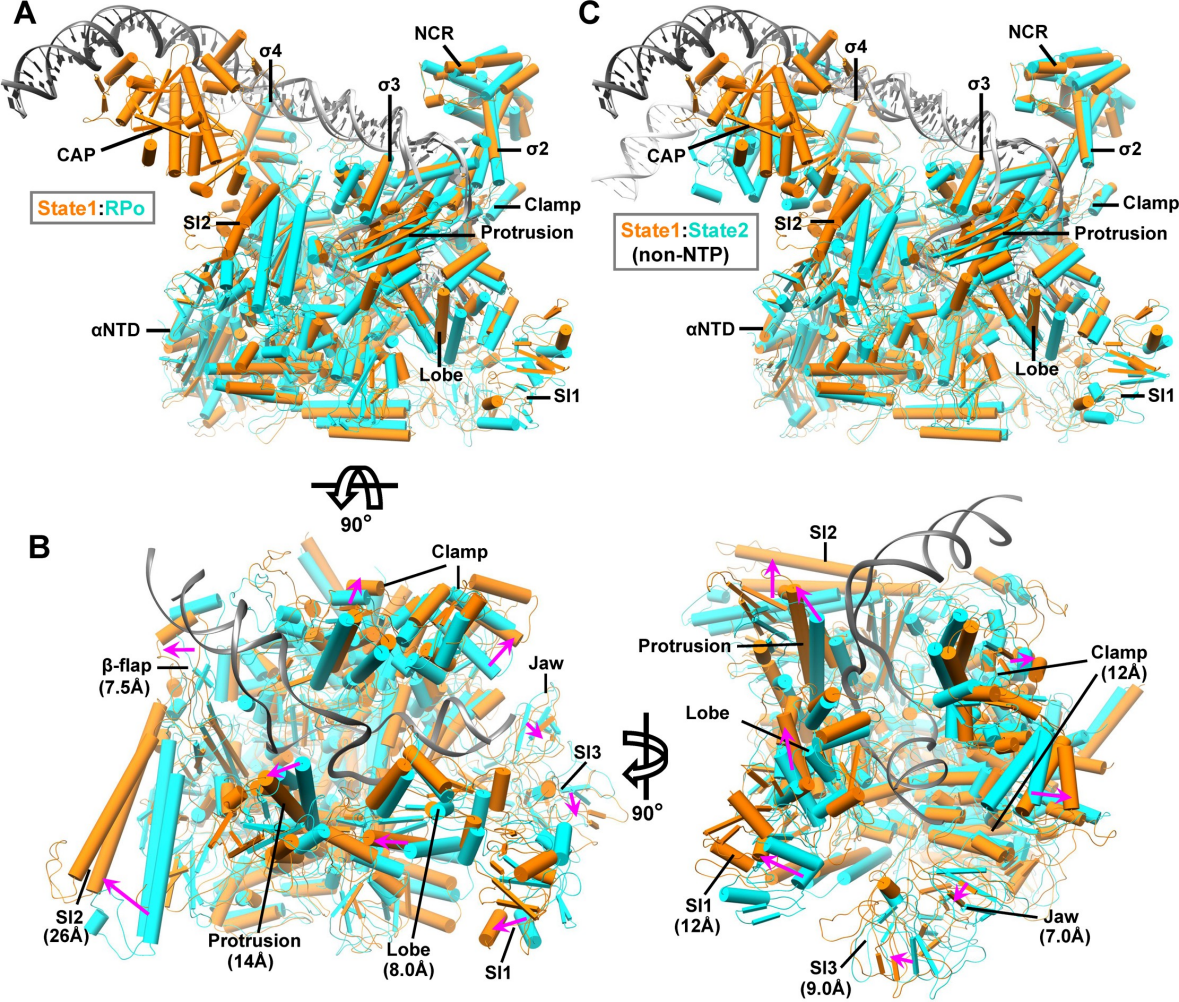
Structural comparisons reveal conformational changes in RNAP. (A) Superimposition of the state 1 CAP-TAC with the previous RPo (PDB 6CA0) [21] via the σ2 and σ3 domains of σ^70^. The components are shown in pipes and planks representation. The state 1 and RPo structures are shown in orange and cyan, respectively, except for dark gray (state 1) and light gray (RPo) DNAs. (B) The close-up views of the main cleft along two directions. The DNA promoter from RPo and all the σ^70^ proteins are omitted for clear representation. The movement directions and maximal distances of the secondary structures in the domains surrounding the main cleft are labeled using magenta arrows and specific values, respectively. (C) Superimposition between the state 1 (orange and dark gray) and state 2 (cyan and light gray) CAP-TAC without RNA transcript via the σ2 and σ3 domains of σ^70^. CAP-TAC, CAP-dependent transcription activation complex; NCR, non-conserved region; NTP, nucleoside triphosphate; PDB, Protein Data Bank; RNAP, RNA polymerase; RPo, RNAP-promoter open complex; SI1, sequence insertion 1; αNTD, amino-terminal domain of the alpha subunit.

The structures of state 1 and state 2 CAP-TACs without NTP incubation show the formation of an RPo with a DNA bubble ranging from −11 to +3, as observed in our previous structure [17], and differing by the position of the CAP dimer, the upstream DNA path, and the conformation of all the RNAP subunits. Remarkably, the state 1 structure of CAP-TAC exhibits an RNAP architecture distinct from the one observed in the previous cryo-EM structure of *E*. *coli* RPo in the absence of CAP binding [21] (Fig 2A). Compared to the previous RPo structure, all the subunits of RNAP in the state 1 structure display substantial conformational changes, especially the domains around the main cleft (Fig 2B). The secondary structures in the β-flap, protrusion, lobe, sequence insertion 1 (SI1), and SI2 domains of the β subunit of RNAP shift to maximum distances of 7.5 Å, 14 Å, 8.0 Å, 12 Å, and 26 Å, respectively. The movements of those in the clamp, jaw, and SI3 domains of the β′ subunit of RNAP reach to maxima of 12 Å, 7.0 Å, and 9.0 Å in distance, respectively. These conformational changes lead to a widening of the main cleft accommodating DNA promoter, which might greatly facilitate DNA promoter entering into the cleft and further isomerizing to RPo (Fig 2B). Because the major difference between the two complexes is whether the CAP dimer is present or not, it is likely that these significant conformational changes observed in the structure of state 1 CAP-TAC might result from CAP binding, besides the possibility of naturally occurred expansion of the main cleft.

Previous biochemical study has shown that in the class-II activation, the interaction between the activating region 2 (AR2) of CAP and the amino-terminal domain of the alpha subunit (αNTD) of RNAP would increase the rate constant for isomerization from the closed to open complexes and therefore suggested AR2-αNTD interaction would either trigger an allosteric change of RNAP or stabilize the intermediate states between the closed and open complexes [11]. Our observations revealed one possible on-pathway intermediate and provided structural support for this proposal. Clamp dynamics has been proposed to be significant in facilitating DNA template melting [22,23]. The opening and close of the clamp domain were apparently observed in prokaryotic and eukaryotic RNAP complexes [24–27]. The narrow and wide statuses of the main cleft have been reported from the multiple structures of *Mycobacterium tuberculosis* RNAP-DNA or RNAP-compound complexes: the clamp-closed status (Protein Data Bank [PDB] 5UHA-5UHG, 5UH5, 5UH6, 5UH8, 5UH9, and 6EDT) [24,28], the intermediate-open status (PDB 6EEC and 6EE8) [24], and the wide status (PDB 6FBV and 6BZO) [29,30]. The two TAC structures (state 1 and state 2) were superimposed with the previous *M*. *tuberculosis* RNAP-DNA complexes (PDB 6EDT, 6EEC, 6EE8, and 6BZO) [24,30], and the results showed that the main cleft in our state 2 architecture displays a similar narrow status as the one in the clamp-closed RPo structure (PDB 6EDT), while the cleft in the state 1 structure is as wide as that in the clamp-open *M*. *tuberculosis* RNAP-DNA complex (PDB 6BZO) (S6A–S6C Fig). Similarly, a contraction and expansion of the main cleft in eukaryotic RNAP I has been reported as well [31]. In summary, all this evidence has supported the presence of the class-II TAC with state 1 architecture observed in this study.

In addition to the unique architecture in the RNAP portion, the relative position of CAP dimer and its bound DNA in the structure of state 1 CAP-TAC, suggested by the density in the cryo-EM map (Fig 1B and S7A Fig), is different from that shown in the state 2 CAP-TAC structure. There is an around 60° switch between the two positions of the CAP-bound DNA (S7B Fig). The new position of the CAP dimer at state 1 extends the canonical contact interface between it and the β subunit of RNAP. The moderate density of the CAP region at the current resolution makes it hard to define the specific interactions with RNAP; however, it is apparent that CAP shifts toward the SI2 region and forms a new interface with the β subunit, which overlaps the interface in the state 2 architecture. The SI2 of the β subunit rotates and shifts by a maximum of 26 Å to interact with CAP, when compared with the state 2 architecture. The β protrusion, β lobe, β′ jaw, and β′ SI3 domains, which all surround the main cleft, make movement due to the pulling force transduced through interactions among these domains. The driving force modulating the β′ clamp domain could be transferred through the σ4 domain or DNA promoter if CAP binding induces this architecture. The RNAP portion of the state 2 CAP-TAC shares a similar architecture as that observed in the previous RPo structure [21], and therefore comparison between the structures of the state 1 and state 2 CAP-TACs also demonstrates the mentioned considerable structural flexibility in RNAP (Fig 2C).

### The conserved interactions in the TACs

The structure of state 2 CAP-TAC without RNA transcript also suggests the interactions among CAP, promoter DNA, and RNAP holoenzyme (S8A and S8B Fig). The positively charged residue (K100) in the AR2 of CAP (residues 19, 21, 96, 100, and 101) [11] is close to the negatively charged residues (D164 and E165) in the 165 determinant (residues 162–165) [11] of the αNTD of RNAP (S8C Fig). The interface between the AR2 of CAP and the β-flap domain of RNAP is the main contact region, primarily involving hydrogen bonds (S8C Fig). The AR3 of CAP (residues 25–27 and 52–58) [11] makes interactions with the 596 determinant (residues 593–603) [32] in the σ4 domain of σ^70^ mainly by salt bridges and hydrogen bonds, as well as with the β-FT by hydrogen bonds. These observed interactions stabilize the activation complex and are consistent with those shown in the previous structural and mutagenesis studies [11,12,15,18,32]. The previously reported recruitment-related AR1 (CAP residues 156–164)–287 determinant (αCTD residues 285–288, 315, and 317) interaction [33,34] was not observed here, which is suggested by the invisibility of αCTD in the map and probably due to flexibility in this region. Crystal structure of *T*. *thermophilus* class-II TAP-TAC did not trap the AR1-287 determinant interaction either, but displayed a new AR4 (corresponding residues 265–301 in *E*. *coli*)–αCTD (corresponding residues 65, 66, 122, and 148–163 in *E*. *coli*) interface [18]. These therefore suggest the high flexibility of αCTD in the activation complex.

### De novo RNA transcript synthesis promotes the conversion of the state 1 to state 2

**Fig 3:**
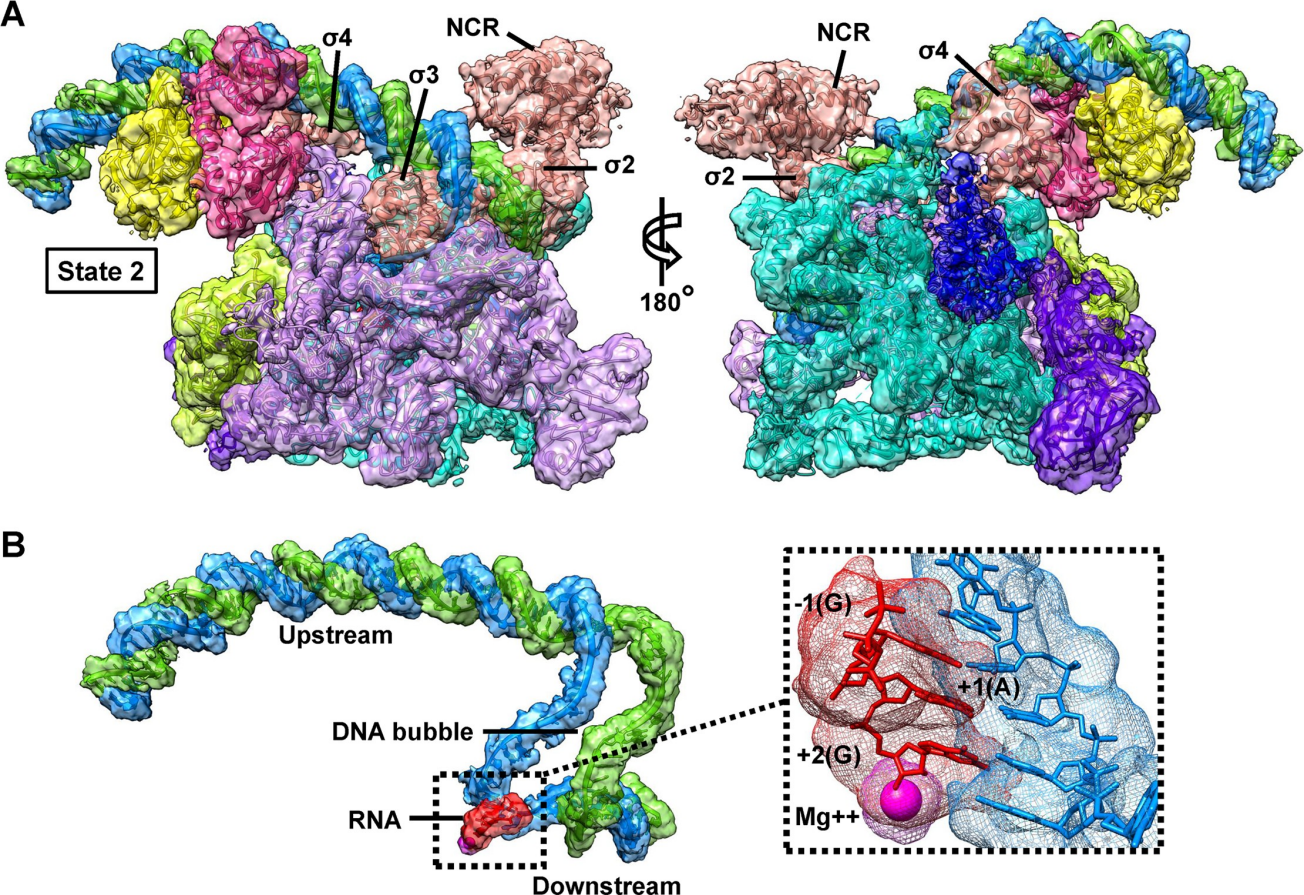
Cryo-EM reconstruction of the class-II CAP-TAC with de novo RNA transcript. (A) Overview of the cryo-EM reconstruction map of the *E*. *coli* class-II CAP-TAC with RNA transcript at 4.4 Å resolution and the state 2 architecture. The color schemes for the split density maps (8 RMS) and the docked components are same as in Fig 1. (B) A close-up view of the promoter DNA scaffold in the complex. The insert is the zoom-in view of the DNA-RNA hybrid region with the magenta Mg^2+^ sphere. A de novo synthesized RNA transcript (3-nucleotide) starting from the −1 position with a GTP residue is displayed. CAP-TAC, CAP-dependent transcription activation complex; cryo-EM, cryo–electron microscopy; NCR, non-conserved region; RMS, root-mean-square.

The cryo-EM density map for the complete class-II CAP-TAC with a de novo RNA transcript was reconstructed at an overall 4.0 Å resolution (S9A–S9D Fig and S1 Table). Further focused classifications and refinements also improved the density for the CAP-binding region and generated a final overall 4.4 Å resolution map allowing us to confidently dock this region (Fig 3A and S9D–S9F Fig). Three-dimensional–FSC analysis suggested no preferred orientation problem in the map (S9G and S10 Figs). The αCTD is invisible in the density map as well, reconfirming the flexibility of this domain in the complex. The density at the active site suggests that a de novo synthesized RNA trinucleotide (GAG) starting from the −1 position is present (Fig 3B), which is consistent with our previous structural studies [17,19]. Local resolution maps showed the relatively weak density on the CAP dimer region (S11 Fig), which is due to its relatively lower occupancy than that of RNAP; however, the intact CAP-RNAP interface is clearly displayed when slightly decreasing the contour level.

The structure of class-II CAP-TAC with de novo synthesized RNA transcript does not capture the state 1 architecture but displays an architecture representing the state 2, and is highly similar to the one of CAP-TAC without NTP incubation (S12 Fig). These observations suggest that de novo RNA synthesis does not affect the overall architecture of the state 2 complex but promotes the conversion of the state 1 to state 2. Subsequent to the clamp opening, which allows DNA to be loaded into in the RNAP active center cleft, promoter unwinding and initial RNA synthesis trigger clamp closure, accounting for the high stability of initiation complexes and the high stability and processivity of elongation complexes [26]. Therefore, it is plausible that the architecture of the state 2 is more stable and resistible than that of the state 1 while resisting DNA scrunch generated during transcription initiation [35,36], and, for that reason, no structure representing the state 1 was observed during RNA transcript synthesis. The overall architecture of the state 2 CAP-TAC is also similar to the structure of the *T*. *thermophilus* class-II TAP-TAC [18] (S13 Fig). No significant difference in the width of the main cleft is observed when superimposing the two structures. However, the relative orientations of the CAP dimer and the domains on the interfaces between CAP and RNAP holoenzyme apparently shift when compared with the *T*. *thermophilus* TAC, probably due to a longer DNA promoter containing the αCTD binding site used in this study.

### Concluding remarks

**Fig 4:**
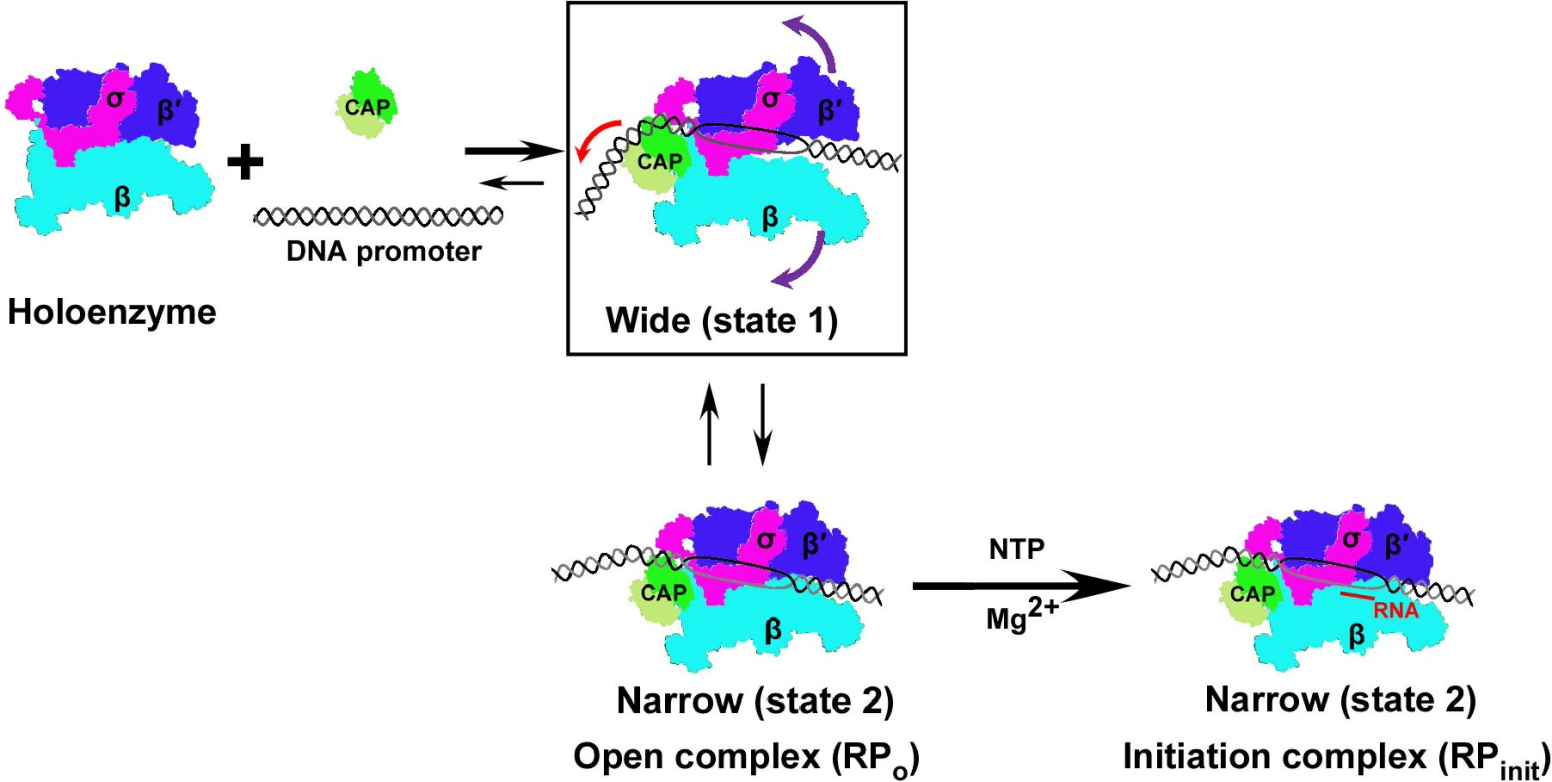
Hypothesized mechanism of transcription activation on class-II promoters. A schematic cartoon model of CAP activating transcription on class-II DNA promoters is presented. When the CAP dimer interacts with RNAP holoenzyme and the class-II DNA promoter, it may either induce conformational changes in RNAP and consequently significantly widen the main cleft to form a state 1 architecture or stabilize the naturally occurred intermediate, which might facilitate the DNA promoter entering into the main cleft. The complex at the state 1 can convert to the one with the state 2 architecture that contains narrow main cleft during the formation of the RPo. With transcription initiation and the synthesis of RNA transcript, all the complexes with different states would convert to the ones with the state 2 architecture. The colored arrows in the rectangle indicate the individual movement directions. CAP, cAMP receptor protein; NTP, nucleoside triphosphate; RNAP, RNA polymerase; RP_init_, RNAP-initiation complex; RPo, RNAP-promoter open complex.

Our structural analyses of intact class-II CAP-TAC in the presence or absence of de novo RNA transcript synthesis displayed three structures with two distinct architectures, which differ by the position of CAP dimer, the upstream DNA path, and the conformation of all the RNAP subunits. These observations may provide insights into how the activation complex undergoes a conformational change process for transcription activation on class-II promoters, in which CAP either induces or stabilizes an on-pathway intermediate (Fig 4).

Based on our structures, we hypothesize that once CAP dimer binds to RNAP holoenzyme and promoter DNA, it first might generate an RNAP-promoter closed complex with CAP at the state 1. The position of CAP in this state 1 closed complex may be different from the observed in this study because the pre-open DNA promoter used here might affect the overall conformation. During the formation of the RPo, the main cleft is widely open, as observed in state 1 architecture, to facilitate DNA promoter entering into it, which may be due to either the CAP binding-induced conformational changes in RNAP or stabilization of the naturally occurred opening by CAP binding. In this process, some of the RPos with the state 1 architecture would transfer into the state 2. With the incorporation of NTP, all the promoter open complexes at the state 1 convert to those with the state 2 architecture. According to this hypothesis, on the class-II promoters, CAP may activate transcription by either inducing or stabilizing the on-pathway intermediate.

## Materials and methods

### Preparation and assembly of *E*. *coli* class-II CAP-TAC

*E*. *coli* RNAP, σ^70^, and CAP proteins were expressed and purified as described [17,19,37,38]. RNAP was then mixed with an excess of σ^70^ and loaded onto a 16/60 Superdex 200 prep grade gel filtration column (GE Healthcare, Chicago, IL) with buffer A (20 mM TRIS pH 8.0, 50 mM NaCl). Fractions containing the σ^70^-RNAP holoenzyme were pooled and concentrated to around 10 mg mL^−1^. To construct a class-II CAP-TAC, we used a synthetic DNA scaffold corresponding to the promoter region between positions −64 and +14 relative to the expected transcription start site (Fig 1A). The synthetic promoter, which contains the αCTD binding site, the CAP protein binding region overlapping the −35 element, the extended −10 motif, and the consensus −10 element, was prepared by annealing the non-template strand to an equal molar amount of the template strand DNA that is complementary to the NT-strand except for a 6-nucleotide discriminator region. The class-II CAP-TACs were assembled by directly incubating the σ^70^-RNAP holoenzyme with a 2-fold molar excess of the preformed DNA promoter and CAP protein in buffer B (20 mM TRIS pH 7.5, 50 mM NaCl, 5 mM MgCl_2_) at 37°C for 10 minutes in the presence of 0.2 mM cAMP, and with or without ATP and GTP (0.2 mM each).

### Cryo-EM sample preparation and data acquisition

The incubated samples were repurified by a Superdex 200 Increase 10/300 GL gel filtration column (GE Healthcare, Chicago, IL) with buffer B to remove the extra CAP protein and nucleic acids. A droplet of 3.5 μL of the repurified class-II CAP-TACs at 1 μM was placed on Quantifoil 1.2/1.3 300 mesh Cu grids (EM Sciences, Hatfield, PA) glow-discharged at 15 mA for 60 seconds. The grid was then blotted for 3 seconds at 4°C under conditions of 100% humidity and flash-frozen in liquid ethane using a Vitrobot mark IV (FEI, Hillsboro, OR). Cryo-EM data were collected on a 300 kV Titan Krios microscope (FEI, Hillsboro, OR) at The Hormel Institute equipped with a Falcon III direct electron detector (FEI, Hillsboro, OR). A 100-μm objective aperture was applied during data collection. Micrographs were recorded in the counted mode at a pixel size of 0.9 Å, with the defocus ranging from −0.8 to −2.6 μm. Recorded at a dose rate of 0.8 e^−^/pixel/second (1 e^−^/Å^2^/second), each micrograph consisted of 30 dose-framed fractions. Each fraction was exposed for 1.5 (CAP-TAC without NTP incubation) or 1.2 (CAP-TAC with NTP incubation) seconds, resulting in a total exposure time of 45 seconds (CAP-TAC without NTP incubation) or 36 seconds (CAP-TAC with NTP incubation) and a total dose of 45 or 36 e^−^/Å^2^. Fully automated data collection was carried out using EPU (FEI, Hillsboro, OR).

### Image processing

Data were processed using cisTEM [39]. A total of 2,369 (CAP-TAC without NTP incubation) or 2,819 (CAP-TAC with NTP incubation) movies were collected. Beam-induced motion and physical drift were corrected followed by dose-weighing using the Unblur algorithm [40]. The contrast transfer functions (CTFs) of the summed micrographs were determined using CTFFIND4 [41]. Particles were then automatically selected based on a matched filter blob approach with the following parameters: maximum particle radius (80 Å), characteristic particle radius (60 Å), threshold peak height (1.5, standard deviation above noise), 30 Å of highest resolution used in picking, and avoiding high variance areas and areas with an abnormal local mean [42]. Initially, 477,218 particles (CAP-TAC without NTP incubation) or 628,208 particles (CAP-TAC with NTP incubation) were picked and extracted to construct a refinement package with 200 Å of the largest dimension and 384 pixels of stack box size. Two-dimensional (2D) classifications [43] were performed using 300-40/8 Å (start/finish, high-resolution limit) data and always without inputting starting references.

For the data of the sample CAP-TAC without NTP incubation, 103 of 200 classes in the first round of 2D classification were manually chosen by discarding the ones containing unsuitable particles and obvious Einstein-from-noise to construct a new refinement package (215,253 particles) and subjected to the second round of 2D classification.

Then, 33 of 200 good classes were picked to construct a new refinement package with 84,537 particles and subjected to Ab-Initio 3D reconstruction [44] to generate an initial 3D model using 20–8 Å resolution data. This initial 3D model was set as the starting reference for the further 3D Auto refinement. FSC at a criterion of 0.143 reported a 4.3 Å resolution for the map outputted from Auto refinement [45]. The flexible and disordered CAP-binding region urged us to perform a focused classification (3 classes) and refinement using 300–10 Å resolution data and a sphere, which has a 60 Å radius and covers the CAP-binding region, in 3D manual and local refinement. Good classes were obtained after 17 cycles of refinements. The second class (46.42%) was reconstructed on 39,242 particles, suggesting an architecture of state 1 with 4.4 Å resolution, in which the architecture of RNAP core and the locations of CAP dimer and its bound DNA are distinct from those of the one that was previously reported [18,21]. However, there is still minor density in the previously displayed location of CAP dimer. Class 1 shows a similar state at a 6.1 Å resolution, while many more mixed states are present in the CAP-binding region of the map from class 3. Further focused classification and refinement with the classes from the first round of focused classification generated a clearer density map on the CAP binding regions in class 2 and class 3. We then combined the particles in these two classes to run Auto refinement and finally got a clearer density map with 4.5 Å resolution.

We also extended the selection range from the second round of 2D classification. A total of 65 of 200 classes, including 115,593 particles, were chosen to subject to the third round of 2D classification. A total of 36 of 80 classes (84,227 particles) were selected to generate a new 3D model (reverse handedness) by Ab-Initio 3D reconstruction. The new model was then refined to 4.2 Å using 3D Auto refinement, revealing an architecture of state 2. Further focused classification (3 classes) and refinement (10 cycles) with a 40 Å radius of sphere centered at the CAP-binding region and use of 300–10 Å resolution data generated much better density on the CAP-binding region from class 2 (37,456 particles, 44.47%, 4.3 Å).

For the data of the sample CAP-TAC with NTP incubation, 35 of 200 classes (171,254 particles) in the first round of 2D classification were manually chosen to subject to the second round of 2D classification. A final package with 109,437 particles (18 of 50 classes) was constructed to generate an initial 3D model using 20–8 Å resolution data by Ab-Initio 3D reconstruction. The model was refined to 4.1 Å using 3D Auto refinement and then to 4.0 Å with further 3D manual and local refinement, revealing an architecture of state 2. Further focused classification (3 classes) and refinement (11 cycles) with a 60 Å radius of sphere centered at the CAP-binding region and the use of 300–10 Å resolution data generated much clear density on the CAP-binding region from class 2 (33,455 particles, 30.57%, 4.4 Å).

### Structural modelling and refinement

The initial models were generated by docking the previous crystal or cryo-EM structures of the components into the individual cryo-EM density maps outputted from the focused classification and refinement using Chimera [46] and COOT [47], including the *E*. *coli* RNAP core enzyme, the bubble region and the downstream DNA portion from the *E*. *coli* σ^s^-TIC (PDB 5IPL) [19], and the upstream DNA portion and σ^70^ from the model of the class-I CAP-TAC (PDB 6B6H) [17]. The 4.4 Å cryo-EM density map from the sample of CAP-TAC with NTP incubation allowed us to build an RNA trinucleotide (GAG) at the active site starting from the −1 position. The omega subunits in all three cryo-EM density maps are still docked according to the previous RNAP holoenzyme structures, although poor densities suggest their flexibilities and low occupancies. The cryo-EM maps representing the state 1 (4.5 Å) and state 2 (4.3 Å and 4.4 Å), from the focused classifications and refinements, show improved density in the CAP-binding region, ensuring further docking of the whole CAP dimer domain using the models from the structures of the CAP-αCTD-DNA complex (PDB 1LB2) [33] and the class-I CAP-TAC (PDB 6B6H) [17]. In all the three cryo-EM maps, no density allowed us to dock αCTD, although we have designed the DNA binding sequence for αCTD.

The intact models were then real-space refined using Phenix [48]. The final maps were put into a large P1 unit cell (*a* = *b* = *c* = 345.6 Å; α = β = γ = 90°) and structural factors were calculated in Phenix [48]. In the real-space refinement, minimization global and local grid searches were performed with the secondary structure, rotamer, and Ramachandran restraints applied throughout the entire refinement. The final model has good stereochemistry by evaluation using MolProbity [49]. The local resolution maps were estimated and generated by ResMap [50] and MonoRes [51] using the online server (http://scipion.cnb.csic.es/m/myresmap). The split cryo-EM maps were generated using color zone with a 2.0 Å coloring radius in the volume viewer of Chimera [46] and shown based on individual volume root-mean-square (RMS). Three-dimensional–FSCs for assessing directional resolution anisotropy were calculated using the online server (https://3dfsc.salk.edu) [52]. The half map sphericity values of all the three cryo-EM maps in this study are all over 0.8, suggesting no concerns on the preferred orientation problem. Guided by the previously described procedure [53], the map-to-model sphericity values and 3D-FSCs were also calculated using Chimera [46], Relion 3 [54], and the 3D-FSC server at an FSC threshold of 0.5. The statistics of cryo-EM data collection, 3D reconstruction, and model refinement were shown in S1 Table. All the figures were created using Chimera [46].

### Quantification and statistical analysis

Quantification, statistical analysis, and validation are implemented in the software packages used for 3D reconstruction and model refinement.

### Data deposition

The cryo-EM maps and the atomic coordinates generated in this study have been deposited in the Electron Microscopy Data Bank (EMDB; http://www.ebi.ac.uk/pdbe/emdb) and the PDB (http://www.rcsb.org) under the accession numbers EMD-20287 and 6PB5 (the class-II CAP-TAC without RNA transcript at state 1), EMD-20288 and 6PB6 (the class-II CAP-TAC without RNA transcript at state 2), and EMD-20286 and 6PB4 (the class-II CAP-TAC with RNA transcript at state 2).

## Supporting information

S1 Fig Isolation of the class-II CAP-TACs by size-exclusion chromatography. (A) The size-exclusion chromatography profile of the CAP-TAC without NTP incubation is presented. The inserted SDS-PAGE gel verified the presence of the complex. (B) The size-exclusion chromatography profile of the CAP-TAC with NTP incubation and the inserted SDS-PAGE gel visualizing the components are presented. The underlying data of panels A and B can be found in S1 Data. CAP-TAC, CAP-dependent transcription activation complex; NTP, nucleoside triphosphate.
(TIFF)

S2 Fig Cryo-EM images and data processing procedure for the CAP-TAC without RNA transcript. (A) A representative micrograph. (B) Flowchart of the cryo-EM image processing (see Materials and methods). (C-D) Selected 2D classes for structure 1 (C) and structure 3 (D), respectively. (E) Gold-standard FSCs of the maps for structures 1–4. (F-G) Angular orientation distribution of the particles used in the final reconstruction for structure 2 (F, state 1) and structure 4 (G, state 2), respectively. The underlying data of panels E–G can be found in S2 Data. CAP-TAC, CAP-dependent transcription activation complex; cryo-EM, cryo–electron microscopy; FSC, Fourier shell correlation.
(TIFF)

S3 Fig Directional FSC analyses of the CAP-TACs without RNA transcript. (A) Directional half map and map-to-model FSCs with the corresponding sphericity values (left), and half map FSCs along x-, y-, and z-axes (right) for structure 2 (the state 1 CAP-TAC without RNA transcript). (B) Directional half map and map-to-model FSCs with the corresponding sphericity values (left), and half map FSCs along x-, y-, and z-axes (right) for structure 4 (the state 2 CAP-TAC without RNA transcript). The underlying data of panels A and B can be found in S3 Data. CAP-TAC, CAP-dependent transcription activation complex; FSC, Fourier shell correlation.
(TIFF)

S4 Fig Three-dimensional–FSC analyses of the CAP-TACs without RNA transcript. (A-B) Histogram and directional FSC plots of half maps (A) and model-to-map (B) for structure 2 (the state 1 CAP-TAC without RNA transcript). (C-D) Histogram and directional FSC plots of half maps (C) and model-to-map (D) for structure 4 (the state 2 CAP-TAC without RNA transcript). The underlying data of panels A–D can be found in S4 Data. CAP-TAC, CAP-dependent transcription activation complex; FSC, Fourier shell correlation.
(TIFF)

S5 Fig Local resolution analyses of the CAP-TACs without RNA transcript. Local resolution maps along two directions for structure 2—the state 1 CAP-TAC without RNA transcript (A; contoured at 0.6 of the view value of Chimera) and for structure 4—the state 2 CAP-TAC without RNA transcript (B; contoured at 0.8 of view value of Chimera). CAP-TAC, CAP-dependent transcription activation complex.
(TIFF)

S6 Fig Superimpositions between *E*. *coli* class-II TACs and *M*. *tuberculosis* RNAP-DNA complexes. (A and B) Superimpositions of the *E*. *coli* class-II CAP-TAC (orange and dark gray, state 2) with the *M*. *tuberculosis* RNAP-DNA-RbpA/CarD (cyan and light gray, PDB 6EDT) and the *E*. *coli* class-II CAP-TAC (orange and dark gray, state 1) with the *M*. *tuberculosis* RNAP-DNA-RbpA/fidaxomicin (cyan and light gray, PDB 6BZO) via the σ2 and σ3 domains are shown, respectively. PDB 6EDT and 6BZO represent the one with the narrowest and widest cleft, respectively, in the determined *M*. *tuberculosis* RNAP-DNA complexes. (C) Zoom-in views of the superimpositions via the σ2 and σ3 domains (from left to right: state2:6EDT, state1:6EEC, state1:6EE8, state1:6BZO), in which 6EEC and 6EE8 represent the ones with the intermediate width of the main cleft. DNA from TACs and RNAPs from all structures are shown. The results suggest that the observed opening and closing of the main cleft in this study are similar to those shown in the structures of the *M*. *tuberculosis* RNAP-DNA complexes. CAP-TAC, CAP-dependent transcription activation complex; PDB, Protein Data Bank; RbpA, RNA polymerase binding protein A; RNAP, RNA polymerase.
(TIFF)

S7 Fig The new position of the CAP dimer and its bound promoter DNA in the CAP-TAC with the state 1 architecture. (A) The transparent split cryo-EM map for the CAP dimer region and the promoter DNA in the state 1 CAP-TAC is shown (contoured at 8 RMS). RNAP holoenzyme is displayed with a pipes and planks representation. The color schemes are the same as in Fig 1. (B) Superimposition of the promoter DNAs between the state 1 and state 2 (magenta) CAP-TACs without RNA transcript shows an approximate 60° swing between the two positions of the CAP dimer and its bound promoter DNA. RNAP holoenzymes were omitted for clear representation. The CAP dimer of state 2 is shown in gray. CAP-TAC, CAP-dependent transcription activation complex; cryo-EM, cryo–electron microscopy; RMS, root-mean-square; RNAP, RNA polymerase.
(TIFF)

S8 Fig The interactions between CAP and RNAP holoenzyme in the state 2 CAP-TAC without RNA transcript. (A) Overview of the *E*. *coli* class-II CAP-TAC without RNA transcript. The transparent split cryo-EM maps (8 RMS) and the components are shown in the same color schemes as in Fig 1. (B) A close-up view of the promoter DNA in the complex. (C) A close-up view of the interface between CAP and RNAP holoenzyme and the zoom-in views of the three main contact areas. In the structure, the CAP dimer makes contacts with the 165 determinant (residues 163–165) of the αNTD and the β-flap domain (residues 854, 859, 862, 863, and 872) through its AR2 (residues 19, 21, 100, and 101), as well as with the β-FT (residues 903–904 and 908–909) and the 596 determinant (residues 593–603) of σ4 using its AR3 (residues 25–27 and 52–58, respectively). It should be noted that there may be some shifts in the side chains of these residues at the current resolution. AR, activating region; CAP-TAC, CAP-dependent transcription activation complex; cryo-EM, cryo–electron microscopy; RMS, root-mean-square; RNAP, RNA polymerase; αNTD, amino-terminal domain of the alpha subunit; β-FT, β flap tip.
(TIFF)

S9 Fig Cryo-EM images and data processing procedure for the CAP-TAC with de novo RNA transcript. (A-C) A representative micrograph (A), the flowchart of the cryo-EM image processing (see Materials and methods) (B), and the selected 2D classes for structure 5 (C), respectively. (D) Gold-standard FSCs of the maps for structures 5 and 6. (E) Directional half map and map-to-model FSCs and the corresponding sphericity values for structure 6 (the state 2 CAP-TAC with RNA transcript). (F) Angular orientation distribution of the particles used in the final reconstruction for structure 6 (the state 2 CAP-TAC with RNA transcript). (G) Half map FSCs along x-, y-, and z-axes for structure 6 (the state 2 CAP-TAC with RNA transcript). The underlying data of panels D–G can be found in S5 Data. CAP-TAC, CAP-dependent transcription activation complex; cryo-EM, cryo–electron microscopy; FSC, Fourier shell correlation; 2D, two-dimensional.
(TIFF)

S10 Fig Three-dimensional–FSC analysis of the CAP-TAC with RNA transcript. (A-B) Histogram and directional FSC plots of half maps (A) and model-to-map (B) for structure 6 (the state 2 CAP-TAC with RNA transcript). The underlying data of panels A and B can be found in S6 Data. CAP-TAC, CAP-dependent transcription activation complex; FSC, Fourier shell correlation.
(TIFF)

S11 Fig Local resolution map of the CAP-TAC with de novo RNA transcript. Local resolution maps along two directions for structure 6—the state 2 CAP-TAC with RNA transcript (contoured at 0.8 of the view value of Chimera). CAP-TAC, CAP-dependent transcription activation complex.
(TIFF)

S12 Fig Superimposition between the two class-II CAP-TACs at state 2. Superimposition of the state 2 CAP-TAC with de novo RNA transcript (orange and dark gray) with the state 2 CAP-TAC without NTP incubation (cyan and light gray) via the σ2 and σ3 domains of σ^70^ is shown. The inserts are the close-up views of the superimpositions in the main cleft, the DNA promoter, and the CAP-binding region, which suggest the high structural similarity between them. CAP-TAC, CAP-dependent transcription activation complex; NTP, nucleoside triphosphate.
(TIFF)

S13 Fig Superimposition between the *E*. *coli* and *T*. *thermophilus* class-II TACs. Superimposition of the *E*. *coli* class-II CAP-TAC (orange and dark gray, state 2 with RNA) with the *T*. *thermophilus* class-II TAP-TAC (cyan and light gray, PDB 5ID2) via the β subunit is shown. The inserts are the close-up views of superimpositions in the main cleft and the CAP-binding region, suggesting the minor change in the width of main cleft but apparent changes in the orientations of the domains on the interface between the CAP dimer and RNA holoenzyme. CAP-TAC, CAP-dependent transcription activation complex; PDB, Protein Data Bank; TAP-TAC, *T*. *thermophilus* class-II TAP-dependent TAC.
(TIFF)

S1 Table Statistics of cryo-EM data collection, 3D reconstruction, and model building. cryo-EM, cryo–electron microscopy; 3D, three-dimensional.
(PDF)

S1 Data Underlying data for panels A and B in S1 Fig. (XLS)

S2 Data Underlying data for panels E-G in S2 Fig. (ZIP)

S3 Data Underlying data for panels A and B in S3 Fig. (XLSX)

S4 Data Underlying data for panels A–D in S4 Fig. (ZIP)

S5 Data Underlying data for panels D–G in S9 Fig. (ZIP)

S6 Data Underlying data for panels A and B in S10 Fig. (ZIP)

## Abbreviations

AR: activating region
cAMP: cyclic AMP
CAP: cAMP receptor protein
CAP-TAC: CAP-dependent transcription activation complex
cryo-EM: cryo–electron microscopy
CTF: contrast transfer function
EMDB: Electron Microscopy Data Bank
FSC: Fourier shell correlation
NCR: non-conserved region
NTP: nucleoside triphosphate
PDB: Protein Data Bank
RMS: root-mean-square
RNAP: RNA polymerase
RPo: RNAP-promoter open complex
RP_init_: RNAP-initiation complex
SI1: sequence insertion 1
TAP-TAC: *T*. *thermophilus* class-II TAP-dependent TAC
αCTD: carboxyl-terminal domain of the alpha subunit
αNTD: amino-terminal domain of the alpha subunit
β-FT: β flap tip
2D: two-dimensiona
3D: three-dimensional
