# Supplementary figures and images for: Visualization of two architectures in class-II CAP-dependent transcription activation
Source: PLoS Biol. 2020 Apr 20;18(4):e3000706. doi: 10.1371/journal.pbio.3000706 (PMC7192510; doi:10.1371/journal.pbio.3000706)

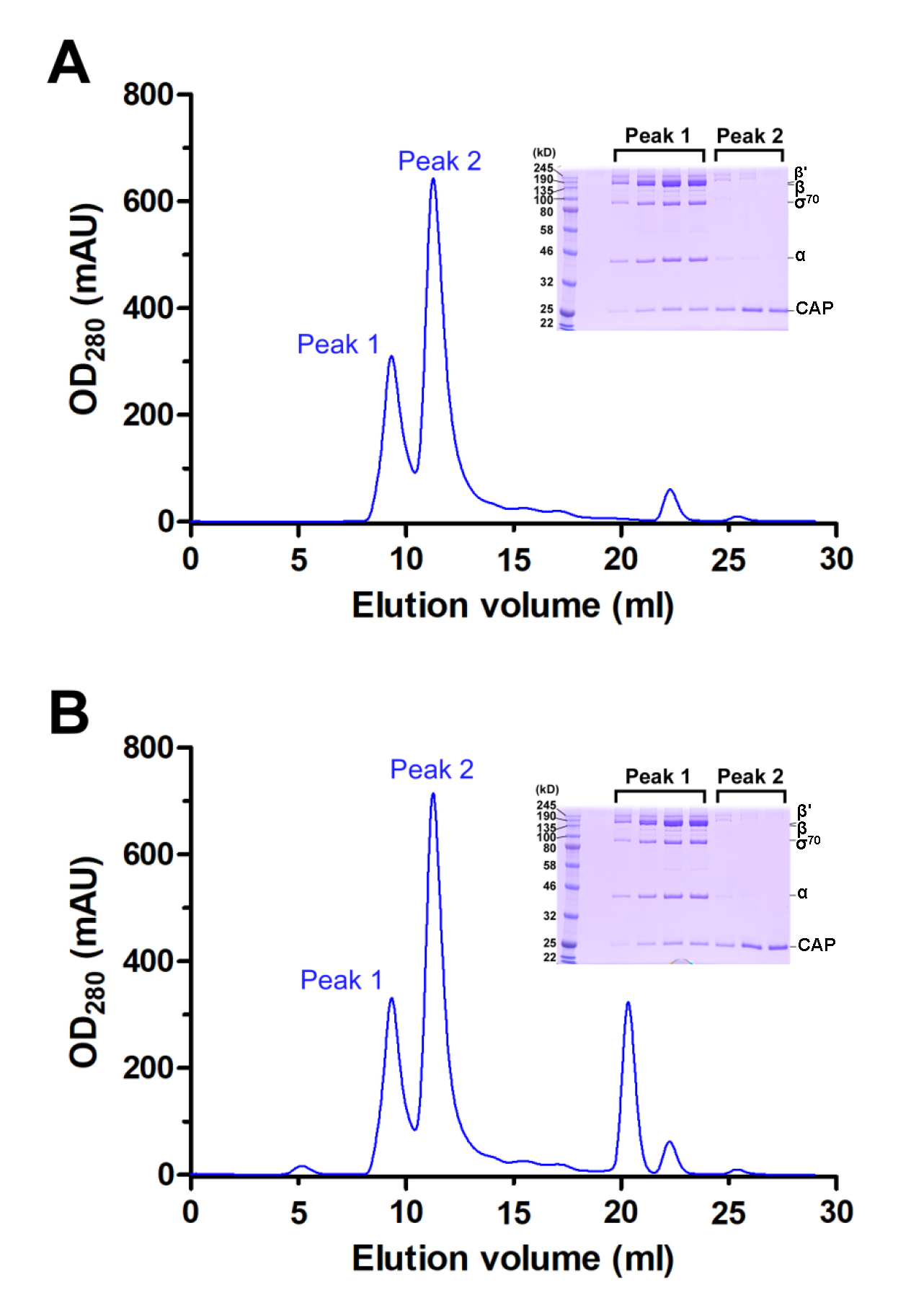

Supplement: S1 Fig — (A) The size-exclusion chromatography profile of the CAP-TAC without NTP incubation is presented. The inserted SDS-PAGE gel verified the presence of the complex. (B) The size-exclusion chromatography profile of the CAP-TAC with NTP incubation and the inserted SDS-PAGE gel visualizing the components are presented. The underlying data of panels A and B can be found in S1 Data. CAP-TAC, CAP-dependent transcription activation complex; NTP, nucleoside triphosphate. (TIFF) [file pbio.3000706.s001.tiff]

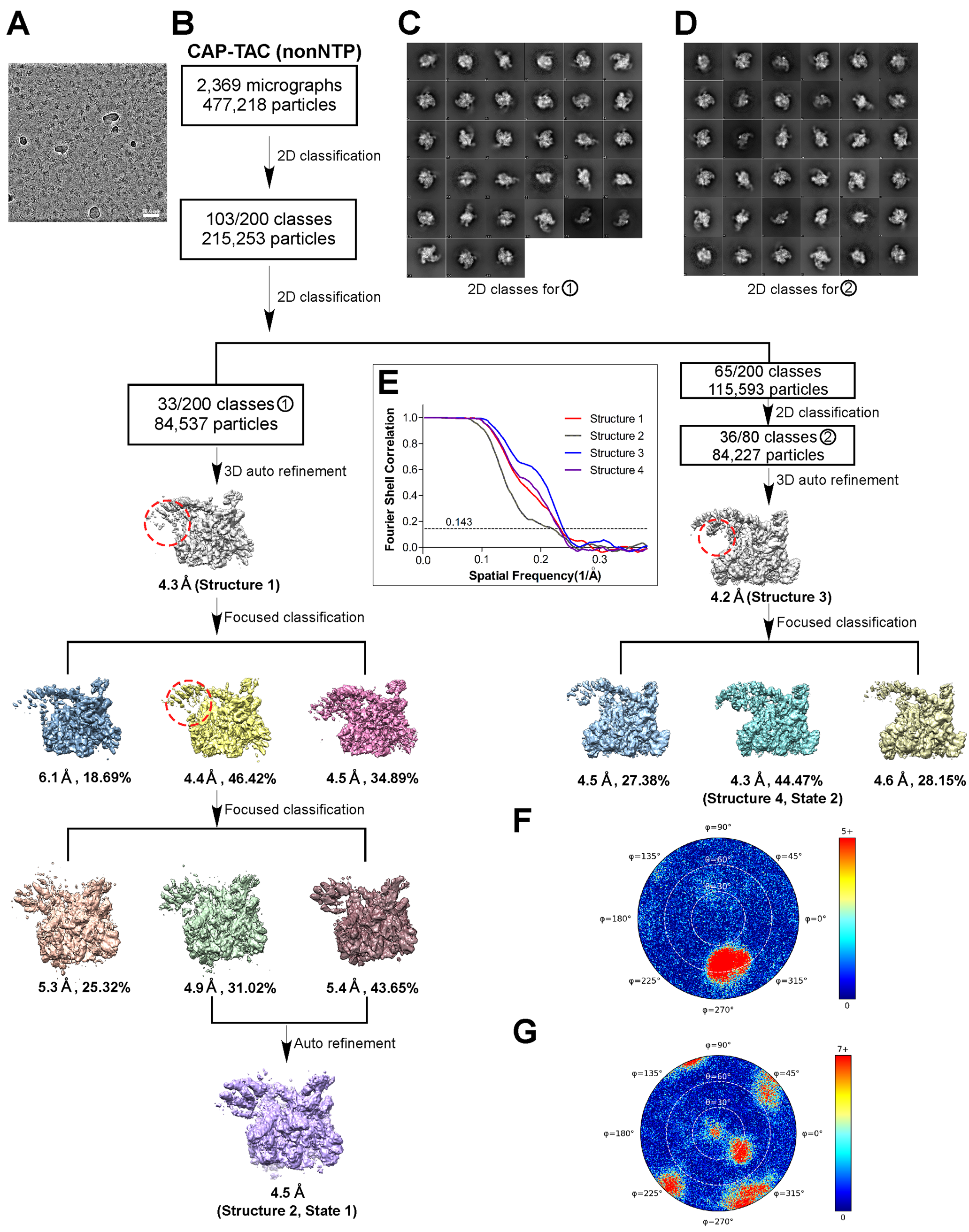

Supplement: S2 Fig — (A) A representative micrograph. (B) Flowchart of the cryo-EM image processing (see Materials and methods). (C-D) Selected 2D classes for structure 1 (C) and structure 3 (D), respectively. (E) Gold-standard FSCs of the maps for structures 1–4. (F-G) Angular orientation distribution of the particles used in the final reconstruction for structure 2 (F, state 1) and structure 4 (G, state 2), respectively. The underlying data of panels E–G can be found in S2 Data. CAP-TAC, CAP-dependent transcription activation complex; cryo-EM, cryo–electron microscopy; FSC, Fourier shell correlation. (TIFF) [file pbio.3000706.s002.tiff]

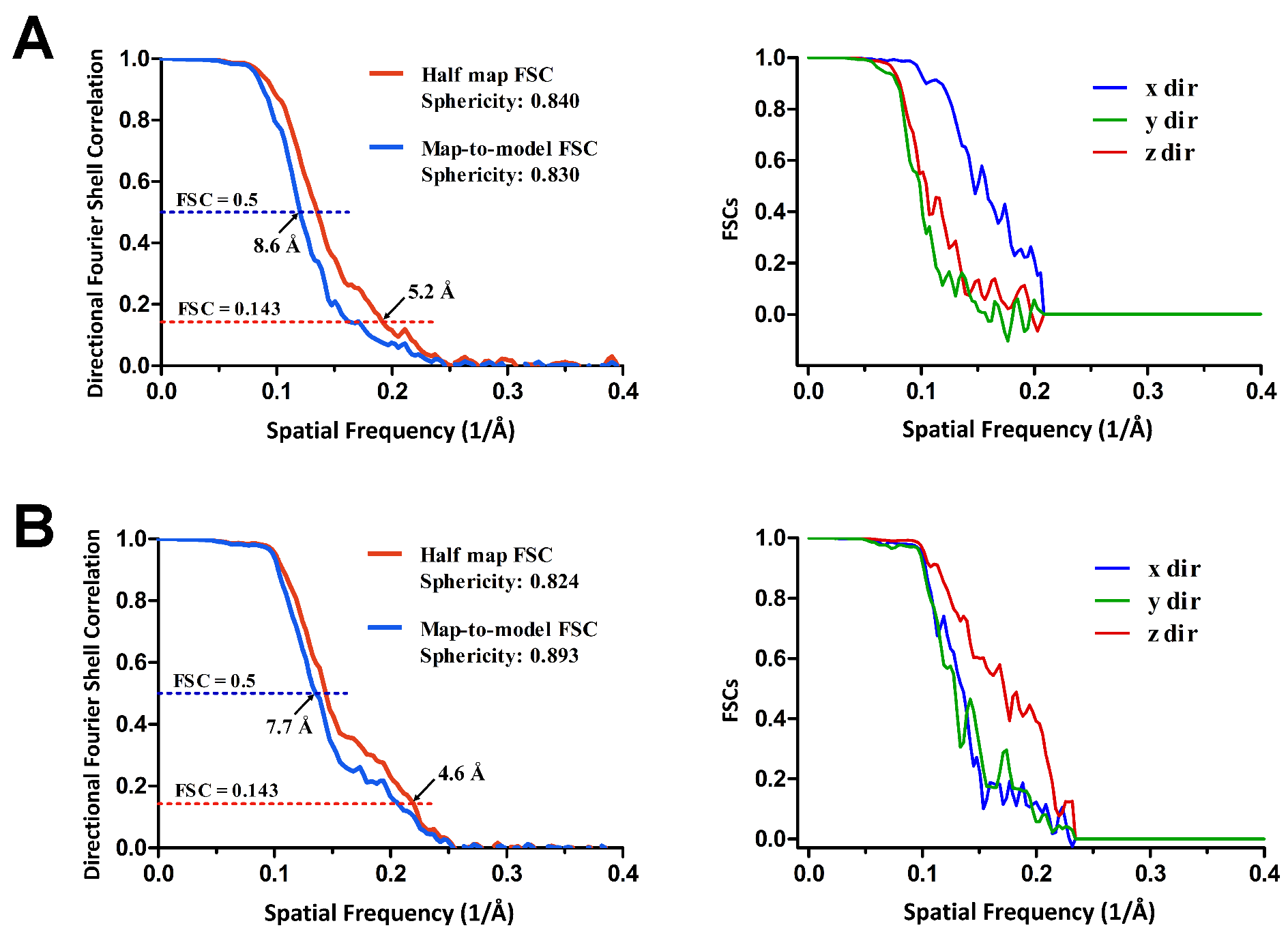

Supplement: S3 Fig — (A) Directional half map and map-to-model FSCs with the corresponding sphericity values (left), and half map FSCs along x-, y-, and z-axes (right) for structure 2 (the state 1 CAP-TAC without RNA transcript). (B) Directional half map and map-to-model FSCs with the corresponding sphericity values (left), and half map FSCs along x-, y-, and z-axes (right) for structure 4 (the state 2 CAP-TAC without RNA transcript). The underlying data of panels A and B can be found in S3 Data. CAP-TAC, CAP-dependent transcription activation complex; FSC, Fourier shell correlation. (TIFF) [file pbio.3000706.s003.tiff]

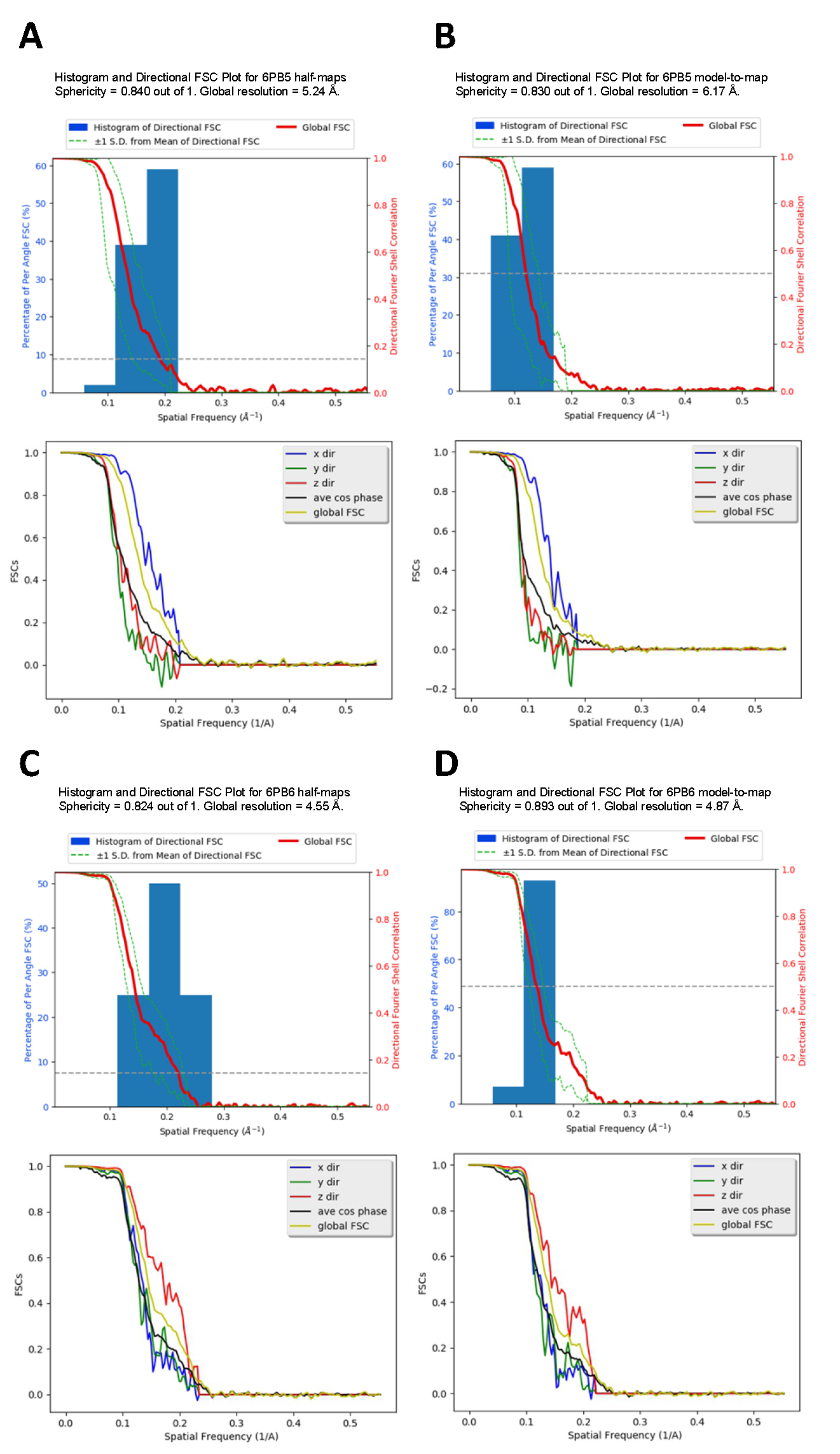

Supplement: S4 Fig — (A-B) Histogram and directional FSC plots of half maps (A) and model-to-map (B) for structure 2 (the state 1 CAP-TAC without RNA transcript). (C-D) Histogram and directional FSC plots of half maps (C) and model-to-map (D) for structure 4 (the state 2 CAP-TAC without RNA transcript). The underlying data of panels A–D can be found in S4 Data. CAP-TAC, CAP-dependent transcription activation complex; FSC, Fourier shell correlation. (TIFF) [file pbio.3000706.s004.tiff]

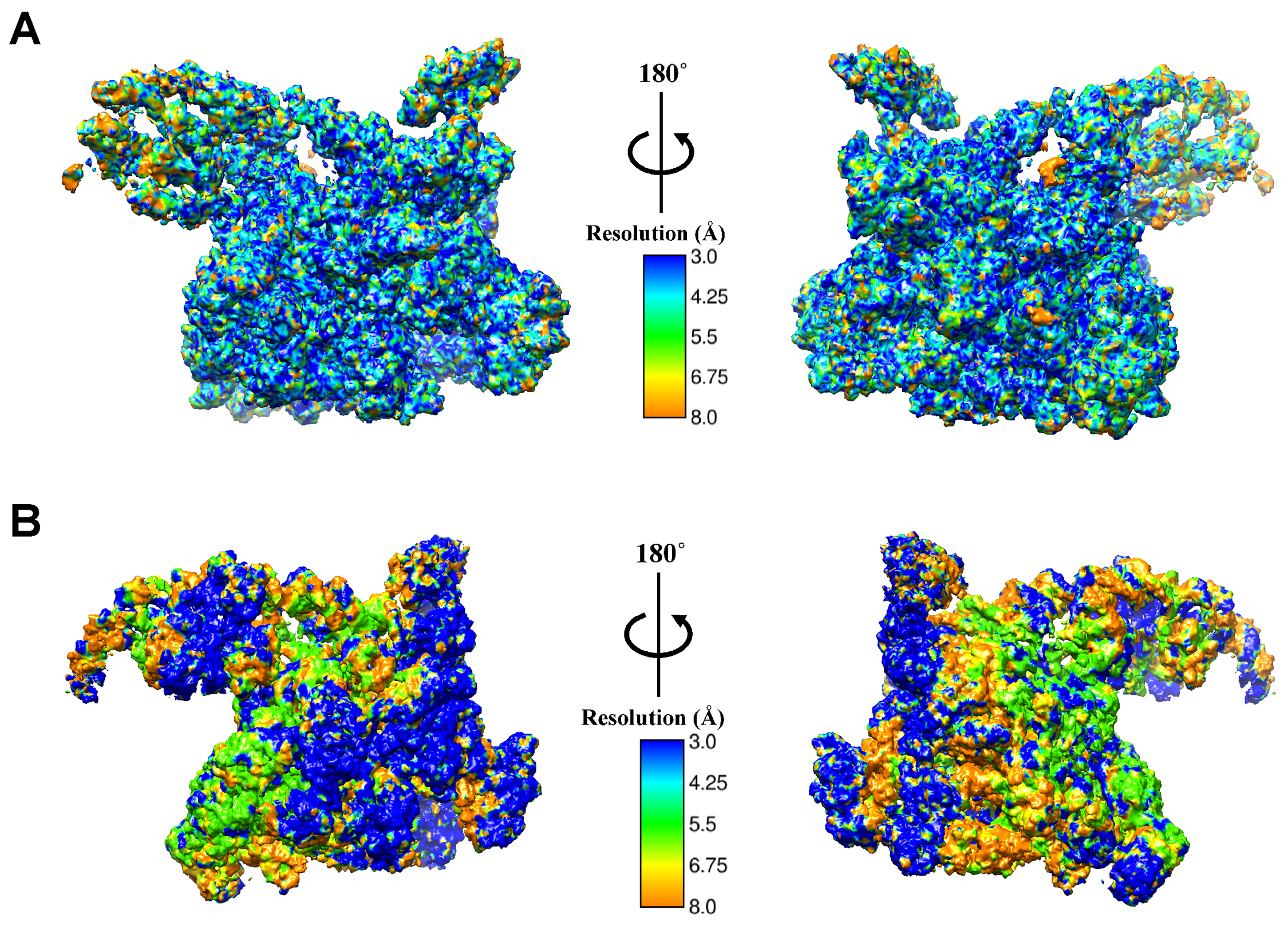

Supplement: S5 Fig — Local resolution maps along two directions for structure 2—the state 1 CAP-TAC without RNA transcript (A; contoured at 0.6 of the view value of Chimera) and for structure 4—the state 2 CAP-TAC without RNA transcript (B; contoured at 0.8 of view value of Chimera). CAP-TAC, CAP-dependent transcription activation complex. (TIFF) [file pbio.3000706.s005.tiff]

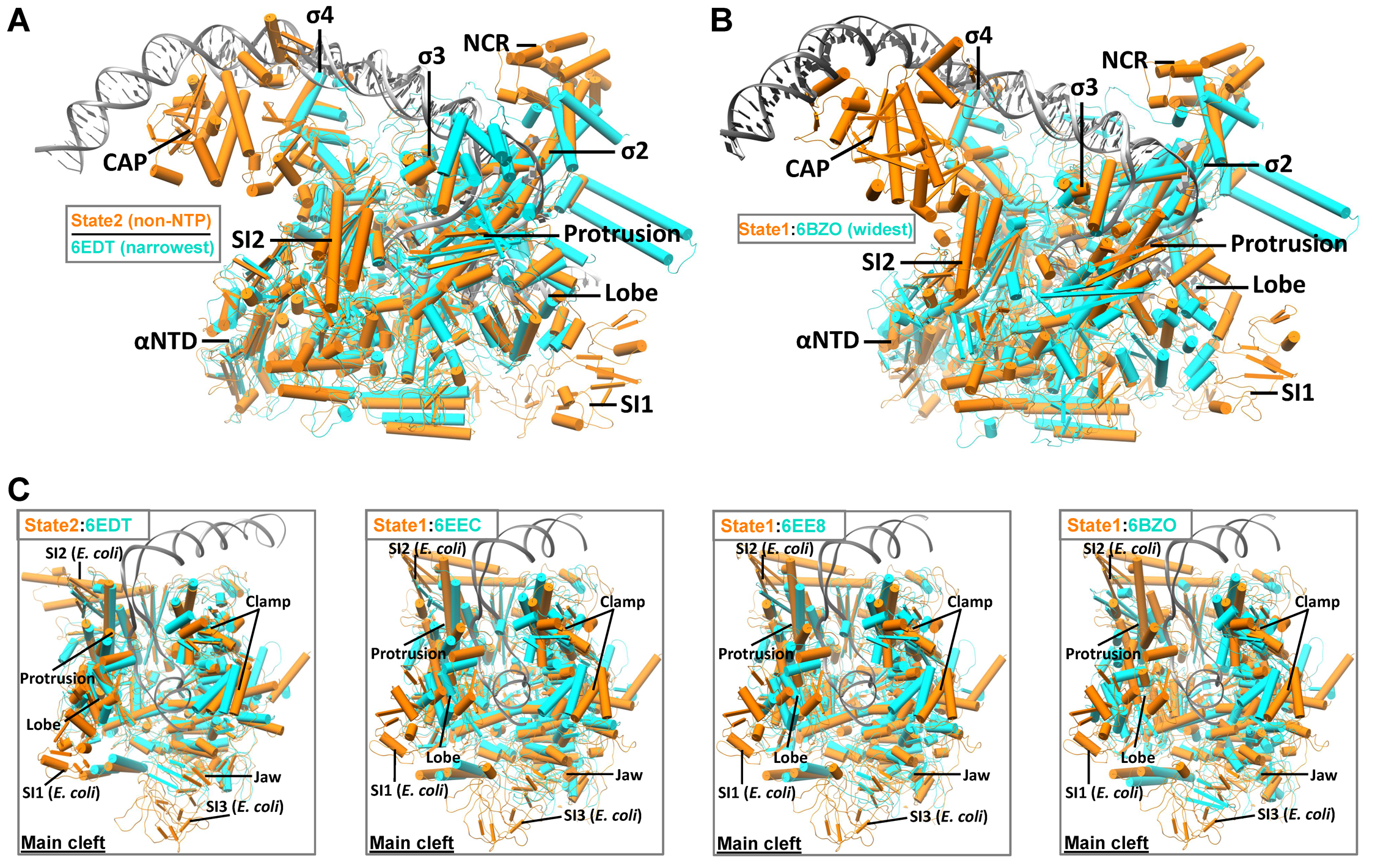

Supplement: S6 Fig — (A and B) Superimpositions of the E. coli class-II CAP-TAC (orange and dark gray, state 2) with the M. tuberculosis RNAP-DNA-RbpA/CarD (cyan and light gray, PDB 6EDT) and the E. coli class-II CAP-TAC (orange and dark gray, state 1) with the M. tuberculosis RNAP-DNA-RbpA/fidaxomicin (cyan and light gray, PDB 6BZO) via the σ2 and σ3 domains are shown, respectively. PDB 6EDT and 6BZO represent the one with the narrowest and widest cleft, respectively, in the determined M. tuberculosis RNAP-DNA complexes. (C) Zoom-in views of the superimpositions via the σ2 and σ3 domains (from left to right: state2:6EDT, state1:6EEC, state1:6EE8, state1:6BZO), in which 6EEC and 6EE8 represent the ones with the intermediate width of the main cleft. DNA from TACs and RNAPs from all structures are shown. The results suggest that the observed opening and closing of the main cleft in this study are similar to those shown in the structures of the M. tuberculosis RNAP-DNA complexes. CAP-TAC, CAP-dependent transcription activation complex; PDB, Protein Data Bank; RbpA, RNA polymerase binding protein A; RNAP, RNA polymerase. (TIFF) [file pbio.3000706.s006.tiff]

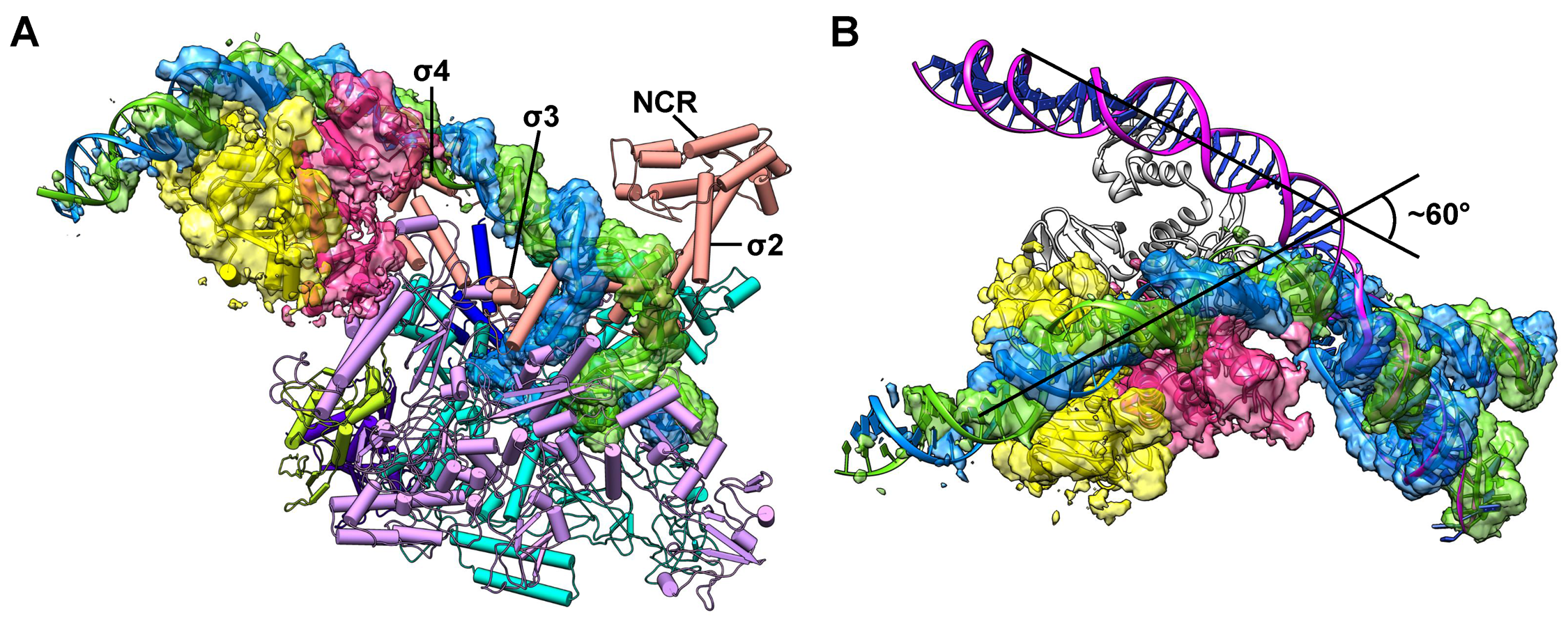

Supplement: S7 Fig — (A) The transparent split cryo-EM map for the CAP dimer region and the promoter DNA in the state 1 CAP-TAC is shown (contoured at 8 RMS). RNAP holoenzyme is displayed with a pipes and planks representation. The color schemes are the same as in Fig 1. (B) Superimposition of the promoter DNAs between the state 1 and state 2 (magenta) CAP-TACs without RNA transcript shows an approximate 60° swing between the two positions of the CAP dimer and its bound promoter DNA. RNAP holoenzymes were omitted for clear representation. The CAP dimer of state 2 is shown in gray. CAP-TAC, CAP-dependent transcription activation complex; cryo-EM, cryo–electron microscopy; RMS, root-mean-square; RNAP, RNA polymerase. (TIFF) [file pbio.3000706.s007.tiff]

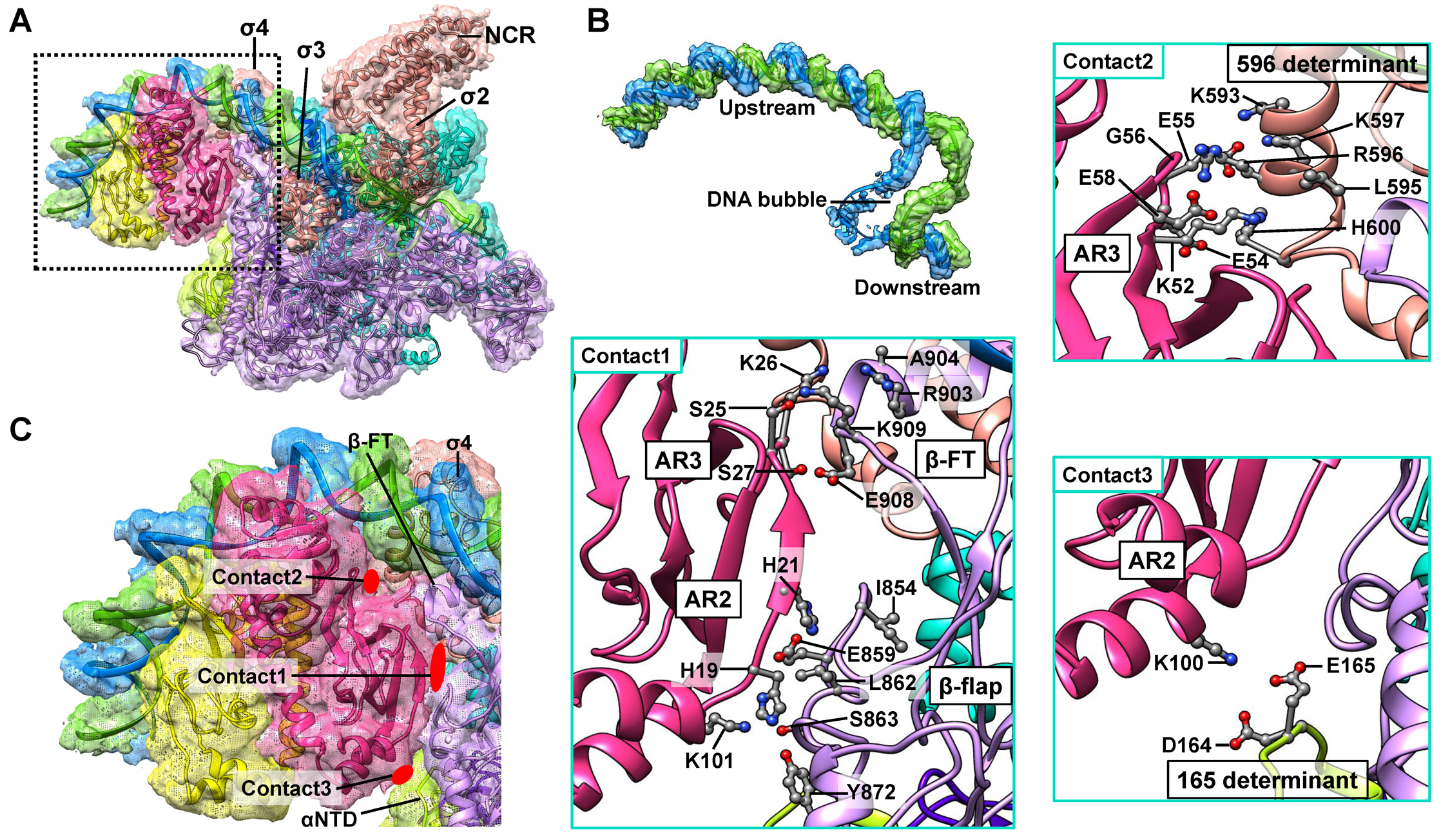

Supplement: S8 Fig — (A) Overview of the E. coli class-II CAP-TAC without RNA transcript. The transparent split cryo-EM maps (8 RMS) and the components are shown in the same color schemes as in Fig 1. (B) A close-up view of the promoter DNA in the complex. (C) A close-up view of the interface between CAP and RNAP holoenzyme and the zoom-in views of the three main contact areas. In the structure, the CAP dimer makes contacts with the 165 determinant (residues 163–165) of the αNTD and the β-flap domain (residues 854, 859, 862, 863, and 872) through its AR2 (residues 19, 21, 100, and 101), as well as with the β-FT (residues 903–904 and 908–909) and the 596 determinant (residues 593–603) of σ4 using its AR3 (residues 25–27 and 52–58, respectively). It should be noted that there may be some shifts in the side chains of these residues at the current resolution. AR, activating region; CAP-TAC, CAP-dependent transcription activation complex; cryo-EM, cryo–electron microscopy; RMS, root-mean-square; RNAP, RNA polymerase; αNTD, amino-terminal domain of the alpha subunit; β-FT, β flap tip. (TIFF) [file pbio.3000706.s008.tiff]

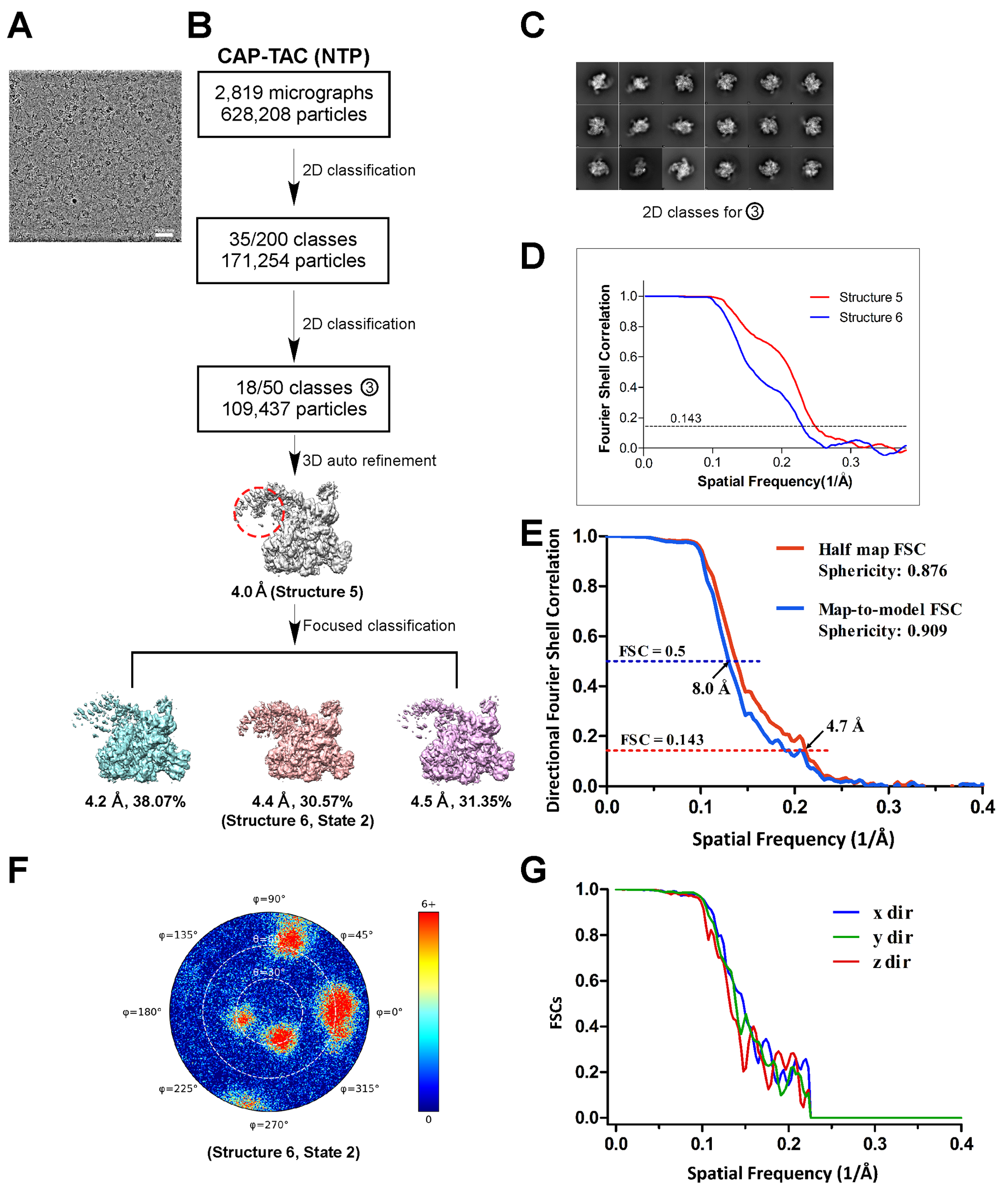

Supplement: S9 Fig — (A-C) A representative micrograph (A), the flowchart of the cryo-EM image processing (see Materials and methods) (B), and the selected 2D classes for structure 5 (C), respectively. (D) Gold-standard FSCs of the maps for structures 5 and 6. (E) Directional half map and map-to-model FSCs and the corresponding sphericity values for structure 6 (the state 2 CAP-TAC with RNA transcript). (F) Angular orientation distribution of the particles used in the final reconstruction for structure 6 (the state 2 CAP-TAC with RNA transcript). (G) Half map FSCs along x-, y-, and z-axes for structure 6 (the state 2 CAP-TAC with RNA transcript). The underlying data of panels D–G can be found in S5 Data. CAP-TAC, CAP-dependent transcription activation complex; cryo-EM, cryo–electron microscopy; FSC, Fourier shell correlation; 2D, two-dimensional. (TIFF) [file pbio.3000706.s009.tiff]

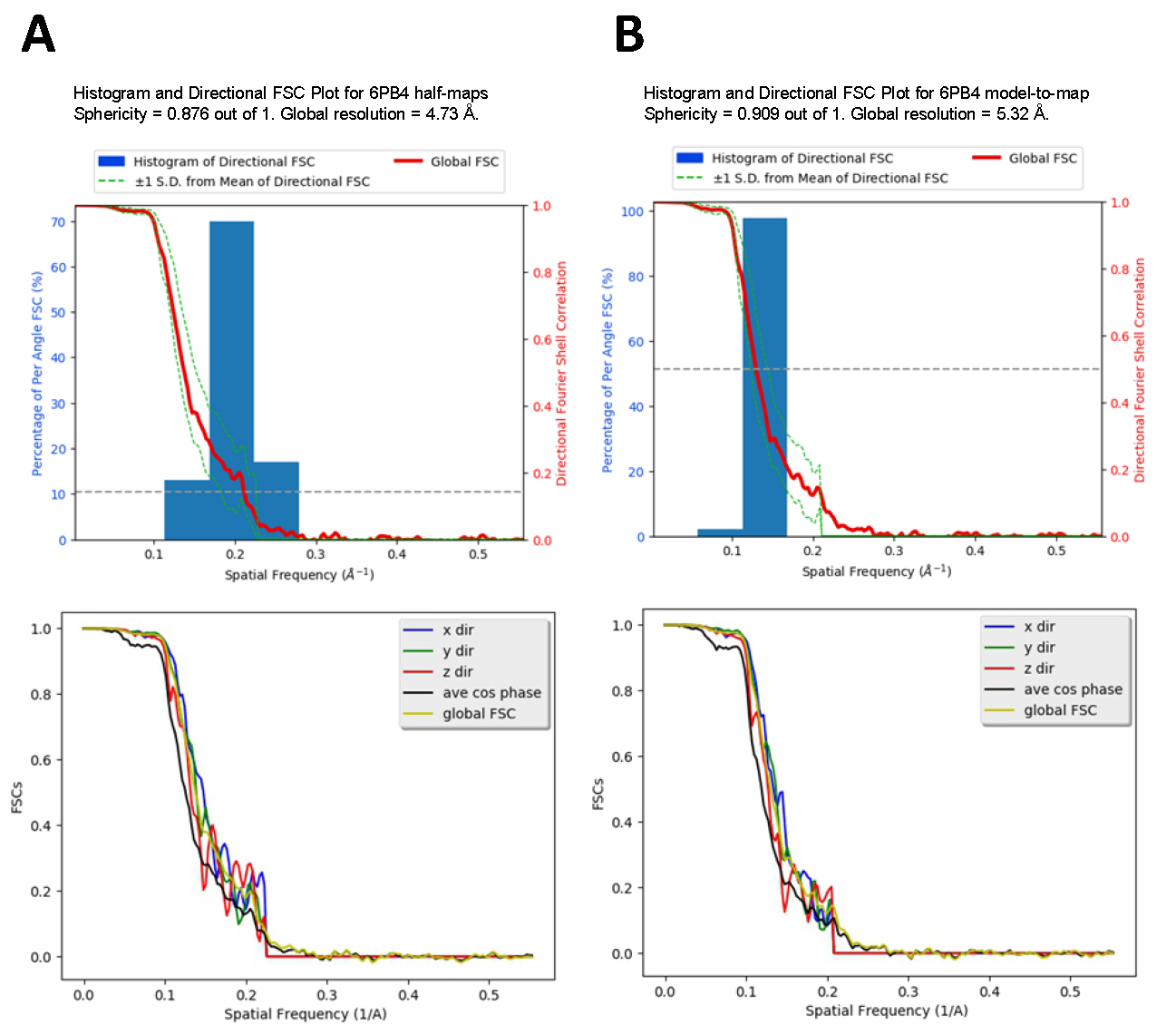

Supplement: S10 Fig — (A-B) Histogram and directional FSC plots of half maps (A) and model-to-map (B) for structure 6 (the state 2 CAP-TAC with RNA transcript). The underlying data of panels A and B can be found in S6 Data. CAP-TAC, CAP-dependent transcription activation complex; FSC, Fourier shell correlation. (TIFF) [file pbio.3000706.s010.tiff]

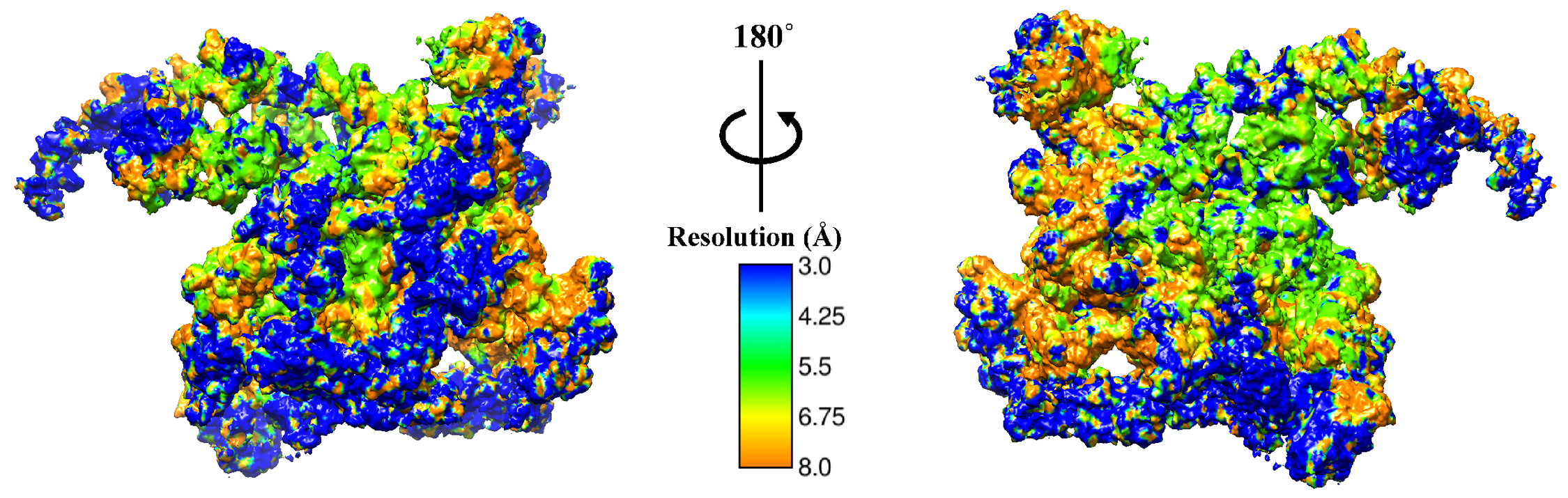

Supplement: S11 Fig — Local resolution maps along two directions for structure 6—the state 2 CAP-TAC with RNA transcript (contoured at 0.8 of the view value of Chimera). CAP-TAC, CAP-dependent transcription activation complex. (TIFF) [file pbio.3000706.s011.tiff]

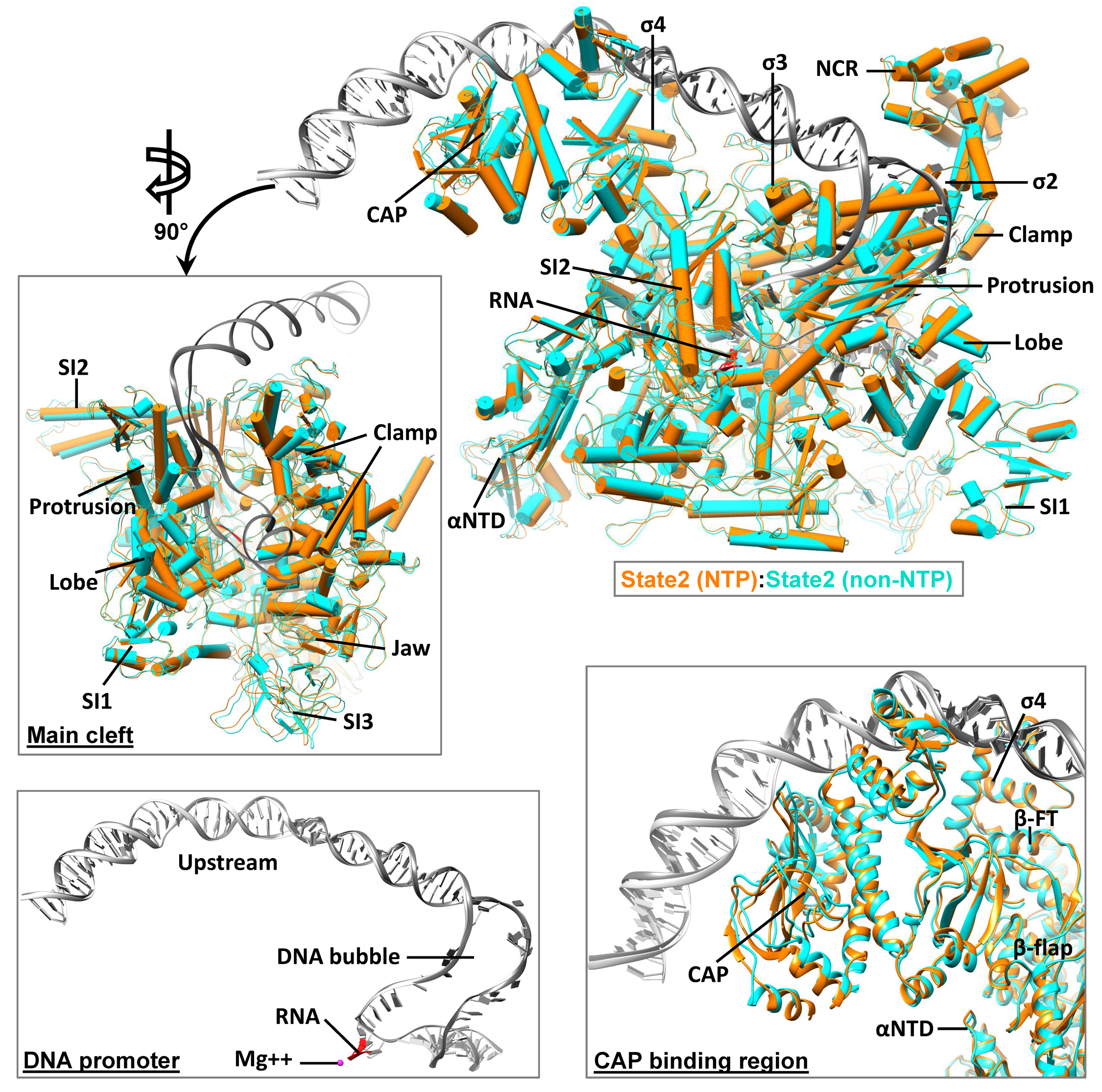

Supplement: S12 Fig — Superimposition of the state 2 CAP-TAC with de novo RNA transcript (orange and dark gray) with the state 2 CAP-TAC without NTP incubation (cyan and light gray) via the σ2 and σ3 domains of σ70 is shown. The inserts are the close-up views of the superimpositions in the main cleft, the DNA promoter, and the CAP-binding region, which suggest the high structural similarity between them. CAP-TAC, CAP-dependent transcription activation complex; NTP, nucleoside triphosphate. (TIFF) [file pbio.3000706.s012.tiff]

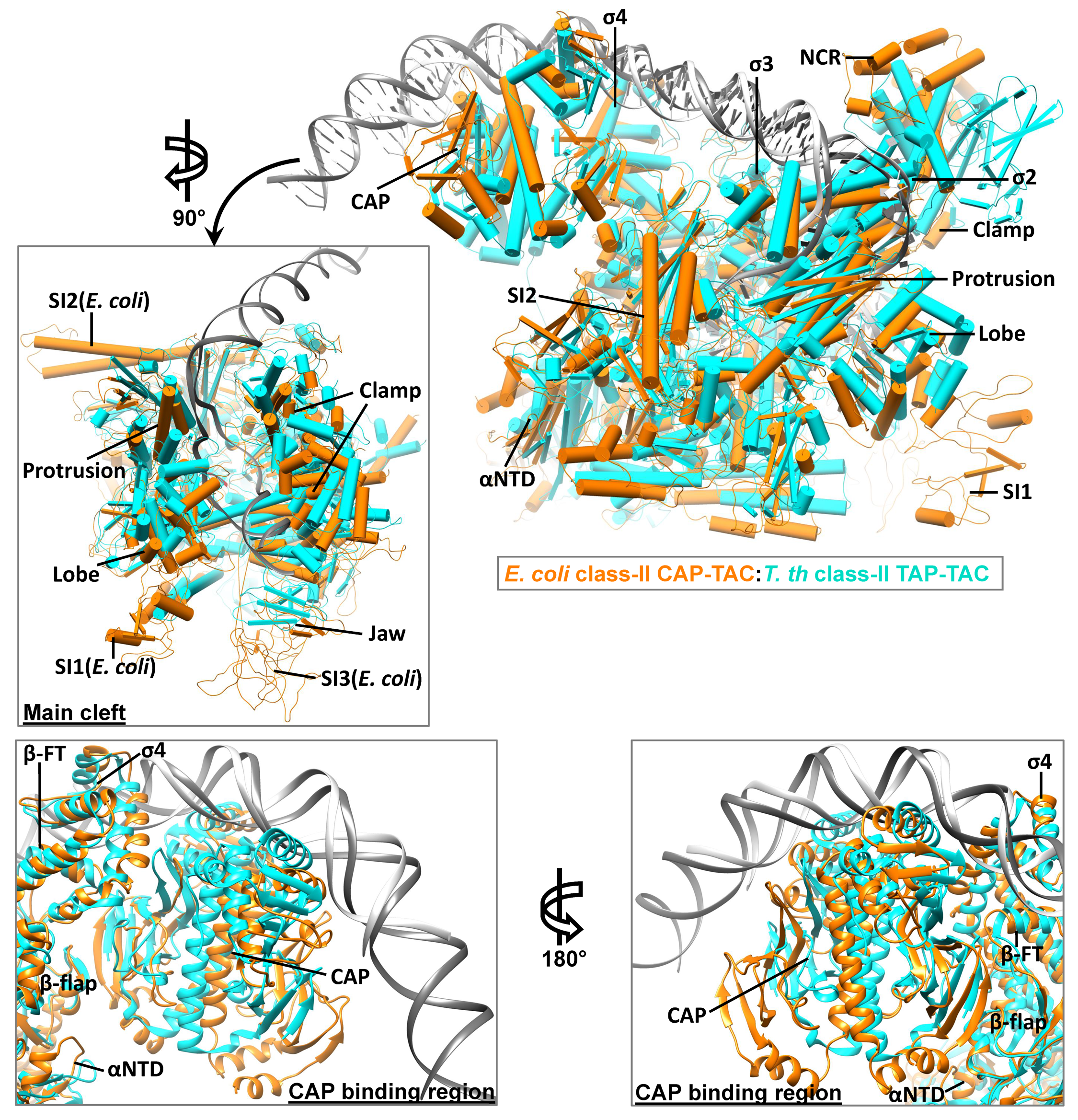

Supplement: S13 Fig — Superimposition of the E. coli class-II CAP-TAC (orange and dark gray, state 2 with RNA) with the T. thermophilus class-II TAP-TAC (cyan and light gray, PDB 5ID2) via the β subunit is shown. The inserts are the close-up views of superimpositions in the main cleft and the CAP-binding region, suggesting the minor change in the width of main cleft but apparent changes in the orientations of the domains on the interface between the CAP dimer and RNA holoenzyme. CAP-TAC, CAP-dependent transcription activation complex; PDB, Protein Data Bank; TAP-TAC, T. thermophilus class-II TAP-dependent TAC. (TIFF) [file pbio.3000706.s013.tiff]
