# Supplementary material for: Visualization of two architectures in class-II CAP-dependent transcription activation
Source: PLoS Biol. 2020 Apr 20;18(4):e3000706. doi: 10.1371/journal.pbio.3000706 (PMC7192510; doi:10.1371/journal.pbio.3000706)
Supplement: S1 Table — cryo-EM, cryo–electron microscopy; 3D, three-dimensional. (PDF) [file pbio.3000706.s014.pdf]

Data collection/processing	CAP-TAC (non-NTP)		CAP-TAC (NTP)
Microscope	Krios		Krios
Voltage (kV)	300		300
Camera	Falcon III		Falcon III
Camera mode	Counted		Counted
Defocus range (μm)	-0.8 ~ -2.6		-0.8 ~ -2.6
Exposure time (s)	45		36
Dose rate (e^-/pixel/s)	0.8		0.8
Magnified pixel size (Å)	0.9		0.9
**Reconstruction (focused maps)**	**State 1**	**State 2**	**State 2**
Software	cisTEM	cisTEM	cisTEM
Symmetry	C1	C1	C1
Particles refined	30296	37456	33455
Resolution (auto-masked, Å)	4.5	4.3	4.4
Access code	EMD-20287	EMD-20288	EMD-20286
**Model Statistics**	**State 1**	**State 2**	**State2**
Number of residues (modeled)	4245	4252	4255
Map CC (Chimera)	0.7382	0.8255	0.8314
MolProbity score	1.89	1.90	1.84
All-atom Clashscore	5.31	5.93	5.03
Cβ deviations	0	0	0
Rotamer outliers	0.23%	0.11%	0.00%
Ramachandran			
Outliers	0.69%	0.29%	0.42%
Favored	87.64%	89.06%	89.19%
RMS deviations			
Bond length	0.008	0.007	0.006
Bond angles	1.221	1.053	0.969
Access code	6PB5	6PB6	6PB4
